# A Novel Egg-In-Cube System Enables Long-Term Culture and Dynamic Imaging of Early Embryonic Development

**DOI:** 10.3389/fphys.2022.893736

**Published:** 2022-05-12

**Authors:** Mohit Dave, Joshua Levin, Seth Walter Ruffins, Yuki Sato, Scott Fraser, Rusty Lansford, Tomohiro Kawahara

**Affiliations:** ^1^ Department of Radiology and Developmental Neuroscience Program, The Saban Research Institute, Children’s Hospital Los Angeles, Los Angeles, CA, United States; ^2^ Department of Stem Cell Biology and Regenerative Medicine, Broad-CIRM Center, Keck School of Medicine, University of Southern California, Los Angeles, CA, United States; ^3^ Department of Anatomy and Cell Biology, Graduate School of Medical Sciences, Kyushu University, Fukuoka, Japan; ^4^ Translational Imaging Center, University of Southern California, Los Angeles, CA, United States; ^5^ Department of Biological Sciences, Division of Molecular and Computational Biology, University of Southern California, Los Angeles, CA, United States; ^6^ Department of Biological Sciences, Dana and Dornsife College of Letters, Arts, and Sciences, University of Southern California, Los Angeles, CA, United States; ^7^ Department of Radiology, Keck School of Medicine, University of Southern California, Los Angeles, CA, United States; ^8^ Department of Biological Functions Engineering, Kyushu Institute of Technology, Kitakyushu, Japan

**Keywords:** embryo development, egg-in-cube, avian embryo culture, imaging, long term culture, quail (*Coturnix japonica*)

## Abstract

The avian egg is a closed system that protects the growing embryo from external factors but prevents direct observation of embryo development. Various culture systems exist in the literature to study the development of the embryo for short periods of incubation (from 12 h up to a maximum of 60 h of egg incubation). A common flaw to these culture techniques is the inability to culture the unincubated avian blastoderm with intact tissue tensions on its native yolk. The goal of this work is to create a unique novel egg-in-cube system that can be used for long-term quail embryo culture initiated from its unincubated blastoderm stage. The egg-in-cube acts as an artificial transparent eggshell system that holds the growing embryo, making it amenable to microscopy. With the egg-in-cube system, quail embryos can be grown up to 9 days from the unincubated blastoderm (incubated in air, 20.9% O_2_), which improves to 15 days on switching to a hyperoxic environment of 60% O_2._ Using transgenic fluorescent quail embryos in the egg-in-cube system, cell movements in the unincubated blastoderm are imaged dynamically using inverted confocal microscopy, which has been challenging to achieve with other culture systems. Apart from these observations, several other imaging applications of the system are described in this work using transgenic fluorescent quail embryos with upright confocal or epifluorescence microscopy. To demonstrate the usefulness of the egg-in-cube system in perturbation experiments, the quail neural tube is electroporated with fluorescent mRNA “in cubo”, followed by the incubation of the electroporated embryo and microscopy of the electroporated region with the embryo in the cube. The egg-in-cube culture system in combination with the “in cubo” electroporation and dynamic imaging capabilities described here will enable researchers to investigate several fundamental questions in early embryogenesis with the avian (quail) embryo on its native yolk.

## Introduction

The main advantage of avian embryos for their use in developmental studies is easy accessibility and the ability to culture the embryos inside the eggshell (in ovo) or on artificial substrates (ex ovo). These embryo culture techniques made it possible for biologists to first describe the normal development of the chicken embryo in detail.

The technique of isolating and culturing embryos on a semi-solid substrate was first started by Waddington (Waddington, 1932) and further improved by Spratt (Spratt, 1947) in a seminal study of amniote form and function. The embryo isolation and ex ovo culture technique developed by New (New, 1955) provided a straightforward technique to culture, manipulate, and observe early avian embryogenesis from primitive streak stages up to Hamburger and Hamilton (HH) stage 12–13 (Hamburger and Hamilton staging system, Hamburger & Hamilton, 1951). Several groups modified New’s culture by placing the isolated embryo between glass/metal rings to primarily extend the period of development observable from primitive streak stages up to HH17 (Gallera and Nicolet, 1961; Jacob, (1971); Jaffee, 1974; Seidl, 1977; Stern and Ireland, 1981). The “Cornish pastry” culture method (Connolly et al., 1995) and its modification MC culture (Nagai et al., 2011) were also derived from New’s culture by adapting to a novel “fold and culture” method in liquid media made from a mixture of albumen and saline. Both these culture methods also worked to achieve better vasculature development up to stage HH18.

The Early Chick (EC) culture method (Chapman et al., 2001) improved upon New’s culture technique by making it quicker and easier to culture chick embryos with more normal tissue tension. The EC culture system enables embryos to be cultured on either dorsal or ventral surfaces from stages HH3-15. However, the use of EC culture in culturing pre-primitive streak embryos remains limited due to low embryo survival rates at those early stages (Chapman et al., 2001). EC culture is still widely used to isolate and culture embryos between late HH3 and HH15, after which some neural and heart morphogenesis defects are often observed (Chapman et al., 2001). One advantage of EC culture over modified New’s culture would be the ease of adapting the culture system to high-resolution microscopy. To enable the culture of pre-gastrulating embryos, New’s culture was modified to isolate and culture pre-primitive streak embryos as early as EGK XII (Voiculescu et al., 2007) [EGK staging system (Eyal-Giladi & Kochav, 1976)]. This work also describes the first few time-lapse imaging experiments to understand the development of the chick embryo in the pre-gastrulation stages. This modified technique enabled isolation of pre-primitive streak embryos but unfortunately did not overcome the laborious nature of the culture technique inherent to the New’s culture system.

Several whole-yolk culture systems have been developed (Auerbach et al., 1974; Tufan et al., 2004; Borwompinyo et al., 2005; Yalcin et al., 2010) to address the shortcomings of the various ex ovo culture methods. In these culture systems, the embryo remains on top of the intact yolk, and all the egg contents with the albumen are transferred to another container for incubation. The containers varied from a turkey or chicken surrogate eggshell (Nirasawa et al., 1992; Borwompinyo et al., 2005), a Petri dish (Auerbach et al., 1974), or cling wrap hammocks (Tufan et al., 2004; Yalcin et al., 2010; Schomann et al., 2013; Tahara and Obara, 2014) for culture. Some systems also include sequential culture in multiple containers involving a plastic cup (0–24 h), quail eggshell (24–76 h), and then in a chicken eggshell (until hatching) with a supply of chicken thin albumen for optimal growth (Ono et al., 1994). Embryos in these whole-yolk culture systems have increased accessibility and can be grown until hatching. Most of these techniques are very useful for studying the development of embryos after the vascular system is established while studying early morphogenesis and gastrulation remained challenging to achieve. While some culture systems enabled access to the early development of the quail embryo (Ono et al., 1994), they were unsuitable for direct observation/microscopy due to culture in a non-transparent eggshell after 24 h of embryo growth.

There is a growing need for a culture system that can enable microscopy of early embryos on their native yolk to mimic natural conditions and maintain proper tissue tension when imaging embryo development. One of the first in ovo imaging studies involved tracing the migration of hindbrain neural crest cells using dye injections, keeping the embryo on its yolk in its native egg environment (Kulesa et al., 2000; Kulesa & Fraser, 2000; Kulesa et al., 2010). These studies used a Teflon membrane on the eggshell window to provide optical transparency and maintain humidity around the embryo during imaging. A complication with this in ovo approach is, that the embryo can drift away out of focus due to its natural 3D expansion on the yolk. This lateral movement is not optimal for long-term time-lapse imaging. An ideal imaging and culture system should address concerns for both ex ovo and in ovo systems described above.

We recently established a generalized design method of the artificial egg-in-cube system, focused on optimizing the oxygen permeability through the cube surfaces and culture conditions for the chick embryo starting at day 3 (E3) until day 7 (E7) of development (Huang et al., 2015). We demonstrate normal embryo development in the cube identical to an eggshell with an added advantage of accessibility to the embryo and its vasculature. We standardized the thickness of the Polydimethylsiloxane (PDMS) membrane (the membrane forms the cube’s sides) to enable higher oxygen diffusion yet provide flexibility enough to insert surgical instruments through the sides of the cube for embryo access.

In the work presented here, we customize the egg-in-cube system to the smaller quail egg, coupling it with fluorescent transgenic quail lines to demonstrate the power of the egg-in-cube system to enable long-term culture and high-resolution imaging of avian embryos from egg-laying stages (EGK-X).

## Materials and Methods

### Fabrication of the Cube and Sample Preparation

The fabrication process of the cubic eggshell and the Polydimethylsiloxane (PDMS) membranes are done as in (Huang et al., 2015) with minor modifications (Supplementary Figure S1). Briefly, PDMS membranes (Sylgard 184 Silicone Elastomer Base and curing agent, Dow, # 2646340) were fabricated and cut into the dimensions of the shorter cube (L*B*H: 24*24*18 mm). These membranes were attached to five sides of the cube frame using liquid PDMS as glue and cured on a hot plate (80°C for 20 min) with its top surface kept unwrapped. The cube was tested for leakage and then sterilized with distilled water and 70% ethanol. The contents of a fertilized quail egg are transferred to the cube. The cube is sealed with a high transparency oxygen permeable membrane (High sensitivity stretch membrane, YSI/Xylem Inc., #098095) or a rectangular piece of sterile & clean cling wrap stretched taut on the open surface and held in place with an elastic rubber band. (Supplementary Figure S1).

### Detailed Design for the on-Stage Incubator

We designed a customized box incubator to house the cube that can be connected to a power supply. This box incubator is equipped with a cylindrical holder to house the cube encapsulated by two heaters made from carbon fibers. A USB carbon heater was modified using heat-shrinkable tubes to make the tubular coil structure. One coil was placed at the center of the holder (lengthwise) on its outer rim. The other was attached to the lid of the box to ensure heating at the level of the embryo. The resistance value (heating capacity) of the two heating coils was calibrated and adjusted by changing the length of the heating coils to obtain the same temperature output from both coils. After assembling the heating coils onto the compact incubator, 5 V DC voltage was applied to the heater coils. By using a thermal imaging camera, the heating output of the coils was adjusted to 37–38°C by changing the DC voltage applied. A small plastic water reservoir fit with an electric fan was used to maintain optimal humidity in the incubator system. For the testing and calibration of the environmental conditions in the incubator, a precise micro-TEMP/RH sensor (Sensirion SHT35 flexible, SysCom Corp.) was used to check the incubation conditions before the experiment. A portable temperature and humidity sensor (EEEKit LCD digital thermometer/hygrometer, supplier: Amazon, #B07KBW4W12) was then used to monitor the real-time temperature and humidity fluctuations in the incubator environment if any. The output terminals for this sensor were extended with electrical wires to measure the local environment very close to the embryo in the cube. This customized incubator setup is highly useful for imaging on microscopes not equipped with on-stage incubators (Supplementary Figure S2A).

### Detailed Design for a Custom Incubator for Long Term Embryo Imaging

The bigger custom incubator is modified from a Tupperware container with carbon fiber-based heaters to provide a constant source of heat to maintain the temperature at 37–38°C. A plastic grating platform keeps the cubes at a height above the small water reservoir. A small fan is used to maintain uniform humidity in the environment. In addition to a precise temperature/humidity sensor with a feedback controller connected to a laptop PC, this incubator was also fitted with a portable temperature/humidity sensor to check the conditions visually. Water was added to the container every 24 h using a small port on the side of the incubator without disturbing the cubes.

### Transgenic Quail

The [Tg(hUbC:H2B-cerFP-2A-Dendra2)] quail line (source: Lansford lab, CHLA) ubiquitously co-expresses histone 2B-ceruleanFP (H2B-cerFP) and Dendra2 (Huss & Lansford, 2017). Dendra2 is a photoconvertible green fluorescent protein that converts to its red form after exposure to near-UV light (Gurskaya et al., 2006). The [Tg(hUbC: Membrane-eGFP)] quail line [Kindly provided by Dr. Jerome Gros (Pasteur Institute, Paris, France)] labels the plasma membrane of all cells (Saadaoui et al., 2020). The [Tg(PGK1:H2B-mCherry)] quail line ubiquitously expresses histone 2B-mCherryFP (H2B-mCherryFP) (Huss et al., 2015a). All animal procedures were carried out following approved guidelines from the Children’s Hospital Los Angeles and the University of Southern California Institutional Animal Care and Use Committees.

### Microscopy and Image Analysis

For the upright confocal imaging modality, time-lapse images were acquired on a Zeiss 780 LSM upright confocal microscope (ZLSM780u) using the W Plan-Apochromat 20x/1.0NA DIC (UV) VIS-NIR M27 75 mm objective or with an upright Olympus MVX10 epifluorescence stereomicroscope with an MVPLAPO x1/0.25NA objective coupled with an Olympus XM10 camera controlled by the Olympus CellSens dimension software (Olympus, RRID: SCR_016238). Imaging on an inverted modality was performed on the Zeiss 780 LSM inverted confocal microscope (ZLSM80i) with ×5/0.16NA, ×10/0.45NA M27, or ×20/0.8NA M27 Plan-Apochromat objectives. Time-lapse images were also acquired using brightfield imaging from an Android Motorola E5 phone camera (original specifications: 13 MP, f/2.0, 1/3.1″, 1.12 µm, PDAF, JPEG resolution: 4368 × 2912). The camera was programmed using a “debug mode” to communicate with a PC laptop. This programming modified the camera resolution to acquire JPEG images at 960 × 720 pixels at 0.7 MP. Images were acquired at regular intervals and stored on the laptop. Images and time-lapse files were processed using the Zeiss Zen (black) software (Zeiss, RRID: SCR_018163), NIH ImageJ (PMID 22743772, NIH, RRID: SCR_003070), and Imaris 9.5 (Bitplane, RRID: SCR_007370).

### Embryonic Staging

Quail embryos were staged based on previously established criteria (Hamburger & Hamilton, 1951; Eyal-Giladi and Kochav, 1976; Ainsworth et al., 2010) with additional detailed descriptions of primitive streak morphology and staging from (Streit & Stern, 2008).

### Time-Lapse Analysis of Embryo Growth in the Cube

The contents of several fertilized wild-type quail eggs were transferred to cubes and set into a customized box incubator (Supplementary Figures S2B, C) for imaging along with intact eggs as controls. A small LED light source was mounted vertically at a suitable distance to provide a constant source of white light on the cubes for bright field imaging. An Android Motorola E5 phone camera was programmed to automatically capture an image at 5-minute intervals. The acquired JPEG images were stored in a computer attached to the phone. Imaging was continued uninterrupted until 96 h of incubation. At 96 h, cubes with normal embryo morphology were repositioned under the camera, and other cubes were discarded. Imaging was continued on these selected embryos until they deteriorated. The acquired JPEGs were converted into TIFF files using ImageJ 1.53. Results obtained from this methodology are described as a part of Section 3.1.

### Testing the Effect of 60% O_2_ on Embryo Growth in the Cube

The contents of several fertilized wild-type quail eggs were transferred to cubes and incubated along with intact eggs as controls in a forced air incubator set at 37°C for 3 days. On day 3, some of the cubes with living embryos were then transferred to a hyperoxia chamber that maintained a constant environment of 60% oxygen (O_2_) along with a set of intact eggs as controls. Embryos in cubes incubated in the forced air incubator and at 60% O_2_ were allowed to incubate and checked every 18–24 h for viability. Non-viable embryos were removed from the cube along with corresponding egg controls and fixed with cold 4% formaldehyde in PBS for 48 h at 4°C. These fixed embryos were then washed in PBS. For staging of these fixed embryos, the length of the 3rd toe and the beak length were measured to stage them according to the Hamburger and Hamilton system (Hamburger & Hamilton, 1951; Ainsworth et al., 2010). The measurement was performed by acquiring images of the 3rd toe and beak placed alongside a plastic ruler on the upright Olympus MVX10 epifluorescence stereomicroscope with an MVPLAPO ×1/0.25NA objective coupled with an Olympus XM10 camera controlled by the Olympus CellSens dimension software.

### Bead Injections Into Embryos

The empty customized box incubator (shown in Supplementary Figures S2A, A’) is pre-equilibrated to 37°C by placing the box in the egg incubator before working with the eggs for 1 h. Wild-type quail embryos were incubated at 37°C in a humidified incubator for 72 h to reach stage E3 (HH18). Contents of these fertilized eggs were gently transferred to several cubes and screened for normal morphology and appropriate staging of embryos was performed. Fluorescent microspheres (1 μm in diameter, Crimson Ex/Em:625/645) (FluoSpheres, ThermoFisher Scientific, #F8816) were diluted 1:1000 into sterile PBS and microinjected using a pulled glass needle into the left lateral vitelline vein along the direction of blood flow. These injected “in cubo” embryos were then transferred back to the 37°C incubator to recover for 30 min. The best injections are first screened on the upright Olympus MVX10 epifluorescence stereomicroscope with an MVPLAPO ×1/0.25NA objective and then transferred to the box incubator on stage and used for dynamic imaging.

### Electroporation of the Quail Embryo “in Cubo”

Multiple [Tg(PGK1.H2B.mCherry)] embryos were transferred to cubes and electroporated with 1 μL of 0.5 μg/μL mRNA, *in vitro* transcribed from the pCS2.membrane.eGFP plasmid (pCS2.membrane.eGFP was a kind gift from Dr. Le Trinh) targeting the midbrain-hindbrain and neural tube regions. Cubes with the electroporated embryos were incubated for 2 h at 37°C in the humidified incubator. After 2 h, electroporated embryos were screened for GFP expression under the upright Olympus MVX10 epifluorescence stereomicroscope and then transferred to the Zeiss LSM inverted confocal microscope stage for imaging. Electroporation conditions: 25 V 50 ms ON/100 ms OFF for 3–5 pulses (Nakamura and Funahshi, 2001).

## Results

### Long Term Culture of the Quail Embryo in the Cube

The egg-in-cube system developed in our previous work was built to hold the contents of chicken eggs (Huang et al., 2015). To adapt it to the quail eggs, we reduced the Polycarbonate (Takiron Co., Ltd, Cat # PCP1609A) cube frame dimensions to a smaller size (L×W×H: 24 × 24 × 18 mm). Our first aim was to characterize the egg-in-cube system and test it for the long-term culture of the quail embryo starting from the unincubated EGK-X stage. Embryo development was much slower, and its morphology was irregular in cubes where the embryo was in close contact with the membrane surface (Supplementary Figure S3). We found that for long-term culture (a few days) in the cube starting from EGK-X, it was essential to have an air gap of ∼5 mm between the surface of the high transparency membrane (top of the cube) and the embryo. Imaging the long-term development (several days) of the embryo on a high resolution upright confocal microscope with the air gap would be restricted to low magnification long working distance objectives like the ×5 and ×10 objectives. Although this air gap did not hamper imaging on upright fluorescent stereoscopes because of their long-distance working objectives.

**FIGURE 1:**
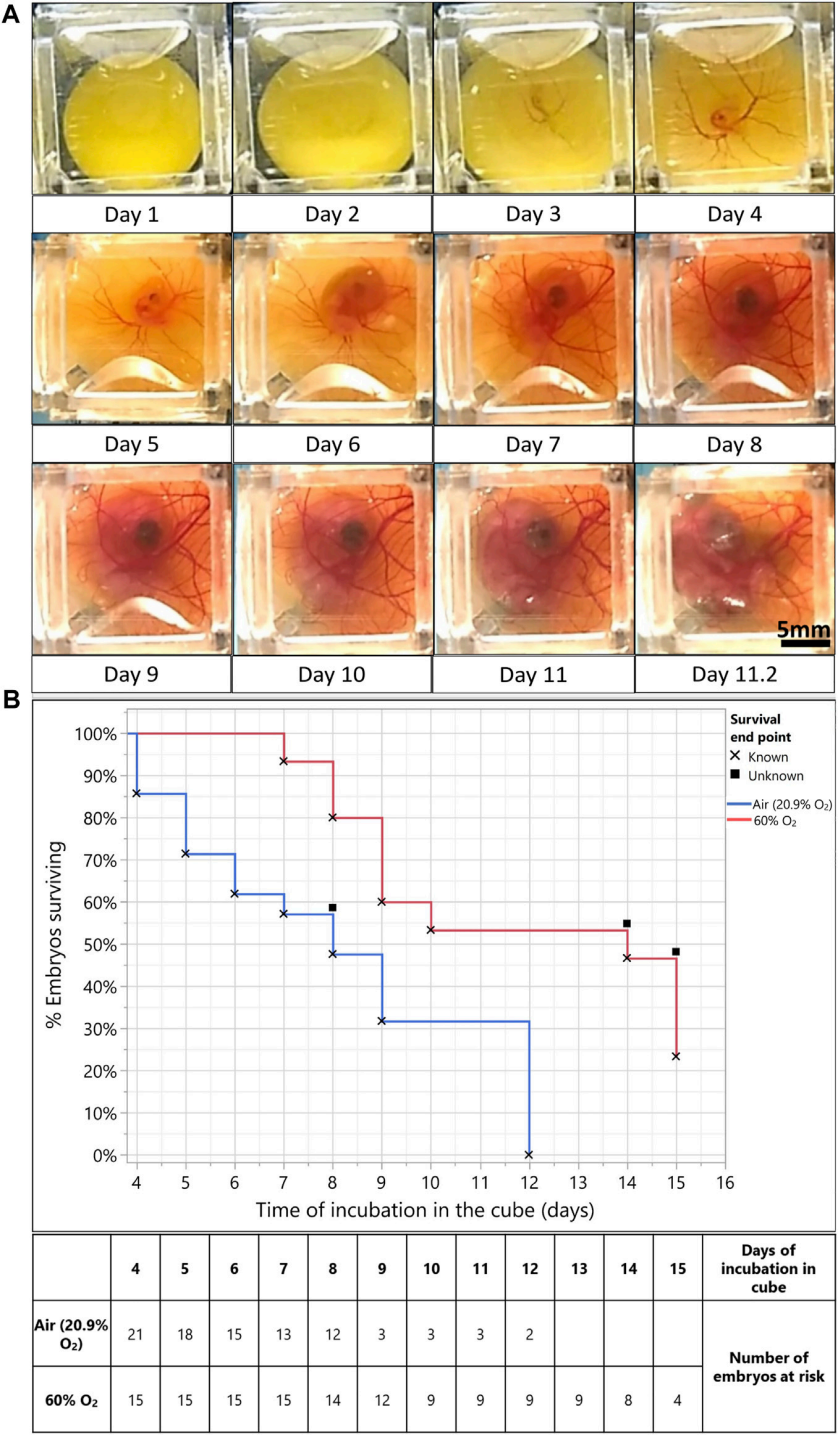
Hyperoxia (60% O_2_) improves embryo survival for long-term culture in the egg-in-cube system. **(A)** Captured frames from live imaging of an EGK-X wild-type quail embryo. The images shown here are acquired using an Android Motorola E5 phone camera taken from the dorsal aspect of the embryo in the cube at 5 min intervals. Images were acquired from EGK-X (E0) to day 4 of development, the lighting and cube were readjusted, and imaging was continued until the embryo started to deteriorate by day 11.25. Raw JPEG files were acquired from the phone and processed into TIFF files using ImageJ. Time frames at approximately 24 h intervals were isolated from these TIFFs to make a representative figure here. The original video file can be seen in Supplementary Video S1. Scale bar = 5 mm. **(B)** Kaplan Meier survival curve plotted between embryos incubated in Air (20.9% O_2,_ blue line, *n* = 21 embryos) and 60% O_2_ (red line, *n* = 15 embryos). Percent embryos survival plotted on the Y-axis vs. Time of incubation in the cube (in days) on the X-Axis. X denotes the death of embryos and filled squares denote the survival endpoint of embryos is unknown/embryos were censored from analysis. Mantel-Haenszel Log-rank test, χ^2^1 = 7.16, *p* < 0.0074.

To test the effect that the revised culture conditions had on the long-term survival of quail embryos in the cube system, we transferred the contents of several EGK-X (unincubated) wild type quail eggs to cubes, introduced an air gap, and set them in a customized box incubator for imaging (See Supplementary Figures S2B, C). Figure 1A shows representative time frames taken from the time-lapse movies of an embryo over 11.25 days (11 days and 4 h) of incubation starting from day 0 (EGK-X, unincubated eggs). The embryo goes through early expansion in the first 2 days, and vasculature develops by the end of day 2 (see Supplementary Video S1). Through days 3–4 of incubation, the blastoderm edges cover the top of the visible yolk, the embryo grows bigger, curves into a C-shaped structure, and now is evident in the brightfield images with its heart beating. By the start of day 5, the allantois is visibly expanding with its vasculature beginning to envelop the embryo. Pigmentation in the eyes is visible; its size increases along with the differentiation of the limbs (∼Day 6).

The head and thorax of the embryo increase in size as the shape of the yolk becomes less spherical due to yolk absorption and metabolism by the yolk sac (Day 7). The chorioallantoic membrane (CAM) vasculature develops further into a dense network of capillaries and vessels on the walls of the cube. The embryo goes through normal development until 11.25 days of incubation; after which its yolk contracts, the vascular system begin to malfunction, and imaging is stopped. Once the imaging was stopped, the embryo was isolated out of the cube for staging. We observed the formation of eyelids, but the embryo had an absence of brown and black pigmentation on its back. Using the presence of these features, we assigned the embryo to a developmental stage of HH35 (∼E8). Thus, the growth of the embryo in the cube was slightly retarded than its expected age at 11 days of incubation.

Embryos cultured in the egg-in-cube system have variable survival times when incubated from EGK-X. In several trials of embryo incubation in the cube, around 23% of embryos died on day 3 of incubation and 34% of embryos survived until 8 days of incubation. The peak in death rate in the initial 3–4 days of incubation has been observed before in literature (Payne, 1919; Riddle, 1930). Embryo death in the first 4 days can occur due to intrinsic factors like gross structural abnormalities and congenital malformations (Byerly 1930), abnormal accumulation of CO_2_ during carbohydrate metabolism, and accumulation of lactic acid (Bohr and Hasselbalch, 1903; Tomita, 1921). One of the important extrinsic factors could be inadequate oxygen supply to the early developing embryo (Riddle, 1930) as it lays down the vascular bed as seen in Figure 1A (Day 4 of incubation). To test this hypothesis, we decided to investigate if supplying a higher amount of oxygen to the embryo from E3 onwards would increase the time of embryo development that can be achieved in the cube and improve the early survival rates.

For this study, several embryos were first transferred to cubes at EGK-X and incubated in a forced air incubator at 37°C for 3 days (*n* = 36 embryos). Some of the cubes (*n* = 15 embryos) with living embryos were then transferred to a hyperoxia chamber maintaining a constant oxygen supply of 60% O_2_ (Stock and Metcalfe, 1984) at 37°C along with matched in ovo controls for long-term incubation. Kaplan-Meier survival curves were plotted for % Percent embryo survival (Y-axis) vs. Time of embryo incubation in the cube (days, X-Axis) to analyze if embryos survived longer due to the added oxygen in their environment. The survival curves (Figure 1B) show that embryos incubated in cubes at 60% O_2_ (*n* = 15 embryos) had significantly better odds of survival (Mantel-Haenszel Log-rank test, χ^2^1 = 7.16, *p* < 0.0074) as compared to those incubated in the air (*n* = 21 embryos, 20.9% O_2_). The median survival time for embryos incubated in cubes at 60% O_2_ (14 days) is also much higher than those incubated in the air (20.9% O_2,_ 8 days). Representative images of embryos incubated at 60% O_2_ vs. those incubated in the air (20.9% O_2_) are shown in Supplementary Figures S6A, B along with the way they were staged.

Thus, with the addition of a hyperoxic environment (60% O_2_), we improved the survival and growth of quail embryos from a median survival time of 8–9 days up to 14 days of incubation. Cultures of all living embryos in the egg-in-cube were terminated at a maximum incubation of 15 days according to the Children’s Hospital Los Angeles (Protocol # 351-16) Institutional Animal Care and Use Committee protocol.

### Imaging Tissue Movements Occurring in a Pre-gastrulation Embryo Using an Upright Fluorescent Stereoscope

**FIGURE 2:**
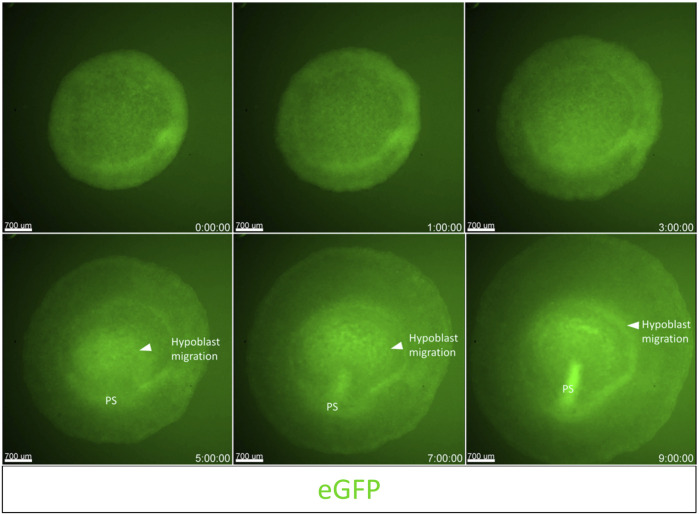
Imaging gastrulation using an upright fluorescent stereoscope. Captured frames from live imaging of an EGK-X [Tg (hUbC.membrane.GFP)] quail embryo. The images shown here are acquired using the upright Olympus stereomicroscope taken from the dorsal aspect of the embryo in the cube at 10 min intervals. Images were acquired from EGK-X (E0) to HH3+ (9.8 h of incubation). Time frames at 1–2 h intervals were isolated from the original TIFF file. The original video file can be seen as Supplementary Video S2 (EGK-X to HH3+). Scale bar = 700 µm.

To demonstrate the capabilities of embryo culture and dynamic imaging using the cube system on an upright fluorescent stereoscope, we used an unincubated EGK-X [Tg(hUbCp.membrane.EGFP)] (Saadaoui et al., 2020) embryo mounted in the cube for imaging. We captured the development of the embryo through the first 10 h of incubation (Supplementary Video S2). The embryo starts expanding slowly through the first 4 h of incubation with polonaise-like movements (Gräper, 1929) in the epiblast accompanied by the classical anterior hypoblast movements (Stern, 1990; Lawson & Schoenwolf, 2003). Both these movements aid in the formation and elongation of the primitive streak towards the anterior end of the embryo, which increases the length of the embryo along the A-P axis as the movie ends at 9 h 50 min. Figure 2 shown here includes representative images from the time-lapse movie highlighting the morphological changes described above.

### Blood Flow Dynamics in an HH15 (E2.5) Wild Type Embryo Injected With Fluorescent Microspheres

Fluorescent microsphere injections have been widely used in mammals to understand myocardial infarctions and regional blood flow measurements (Glenny et al., 1993; Deveci & Egginton, 1999; Goyal et al., 2013). This technique has been extended to chick embryos for the measurement of cardiac output in E10 chick embryos in normal and hypoxic conditions (Mulder et al., 1997; Mulder et al., 1998). Here, we microinjected fluorescent crimson microspheres into the blood circulation of an E3 wild-type quail embryo and imaged the movement of microspheres in smaller capillaries in the extra-embryonic vasculature.

**FIGURE 3:**
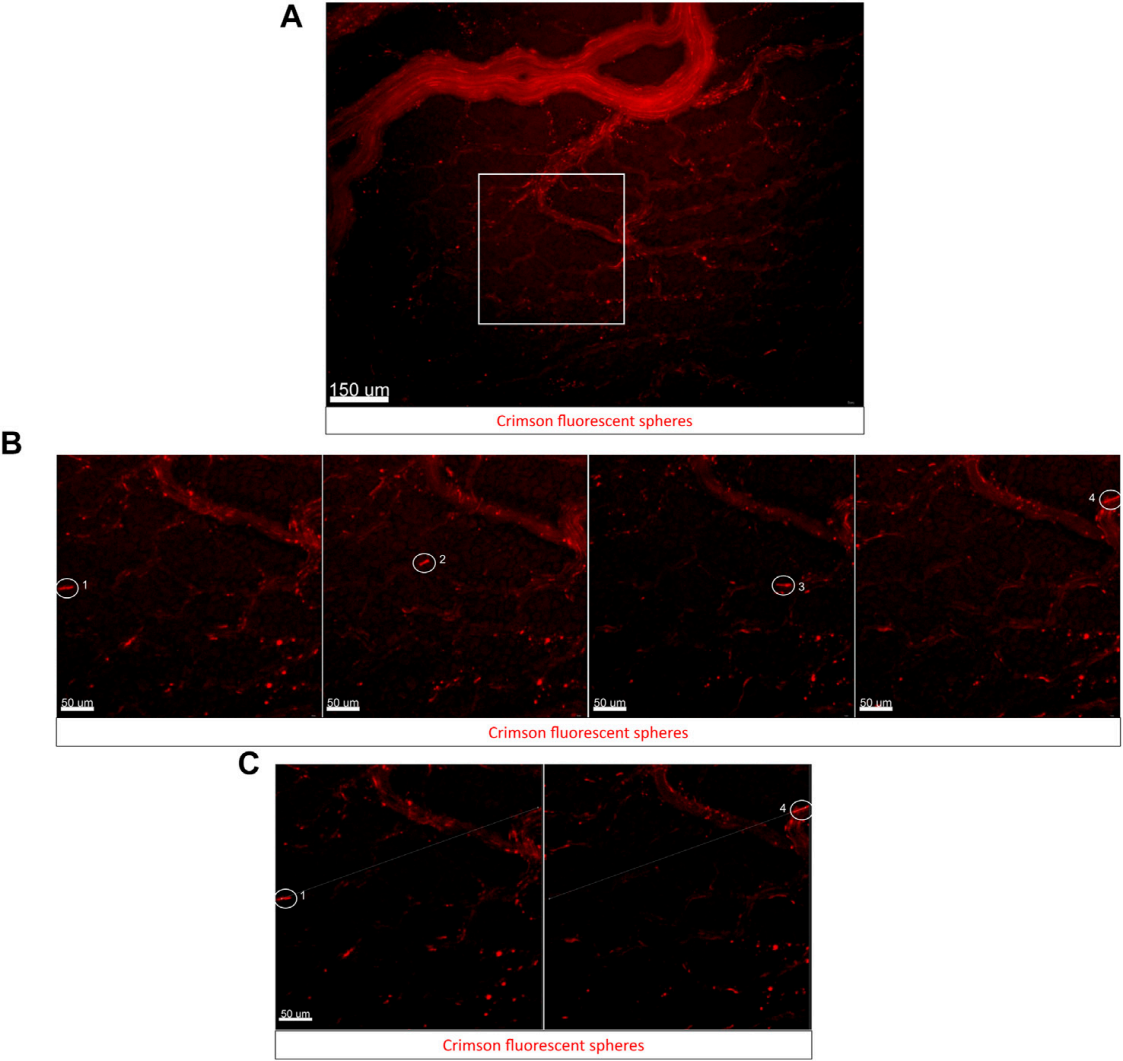
Using fluorescent bead injections to study blood flow dynamics in embryos using the cube on an upright fluorescent stereoscope. **(A)** Captured frame from live imaging of an E3 wild-type quail embryo in the cube. The embryo was microinjected with a small bolus of fluorescent microspheres/beads in the lateral left vitelline vein, the cube was covered with the high transparency membrane and mounted into the custom incubator for imaging. The images shown here are acquired using the upright Olympus stereomicroscope taken from the dorsal aspect of the embryo in the cube at 50 ms intervals. Panel **(A)** shows the representative region of interest (ROI) used for bead tracking analysis. **(B)** Captured frames from the ROI (white square) from **(A)**. The encircled bead moves through the small capillary and is tracked through its path in the vessel here. Representative images from four time frames 50 ms, 450 ms, 900 ms, and 1500 ms from the start of the time-lapse show the bead moving through the vessel (labeled 1–4 in order of frame sequence). **(C)** The first frame and last frame are shown in **(B)** and are used for tracking the displacement of the microsphere through the circulation (Measurement tool in Imaris). The original video file can be seen as Supplementary Video S3A [original time-lapse of region shown in Panel 3A] and Supplementary Video S3B [Zoomed in time-lapse of region shown in Panel 3B].

Within 30 min of injection, fluorescent beads are distributed throughout the embryonic and extra-embryonic circulation. Figure 3A (Supplementary Video S3A) shows the beads circulating in the extra-embryonic blood vessels on the left side of the A-P axis of the embryo. Panels in Figure 3B shows a zoomed-in view of a region of interest from Figure 3A with small capillaries. As a proof of concept for studying regional blood flow using the cube, a cluster of beads (circled in white, Figure 3B) was tracked through the 30 frames in the time-lapse (4 representative frames at 50, 450, 900, and 1500 ms shown in Figure 3B, Supplementary Video S3B). Figure 3C shows the representative initial and final positions of the fluorescent bead tracked in Figure 3B. The average velocity of beads in circulation was found to be 0.55 ± 0.14 mm/s (values are mean ± SEM, *n* = 6 beads tracked).

Fluorescent microbeads have been used by other groups in quail embryos for studying blood flow dynamics, values for blood flow velocity reported by these groups varied significantly in extra-embryonic vasculature regions imaged across different embryos at HH13 (0.3–2 mm/s) depending on vessel diameters and proximity to major blood vessels (Ghaffari et al., 2015). These studies employ an elaborate setup to keep the embryo growing atop its yolk in a Petri dish covered with albumen. The egg-in-cube system will make it much easier to microinject, track, and quantify the velocity of beads in circulation using potentially all possible types of imaging modalities.

### Imaging Cardiac Neural Crest Cell Migration Using Photoconversion of the Dendra2 Protein Using Transgenic Embryos in the Cube

Cardiac neural crest (CNC) cells are pluripotent stem cells that give rise to the pharyngeal arch arteries and cardiac outflow tract (Le Lièvre & Le Douarin, 1975; Kirby et al., 1983). They arise from the dorsal neural tube region between the otic vesicle and the 3rd somite at HH11 and migrate laterally towards the ventral surface of the embryo until at HH17 when they start to appear in the precardiac wall, in regions circumscribing the pharyngeal ectomesenchyme and the anterior cardinal vein (Kirby et al., 1985; Hutson & Kirby, 2007). Confocal imaging of neural crest cell migration was achieved in ovo by (Kulesa et al., 2000; Kulesa & Fraser, 2000). This in ovo imaging technique enabled a long time of observation and had the advantage of keeping the embryo with its proper orientations and tissue tension intact.

**FIGURE 4:**
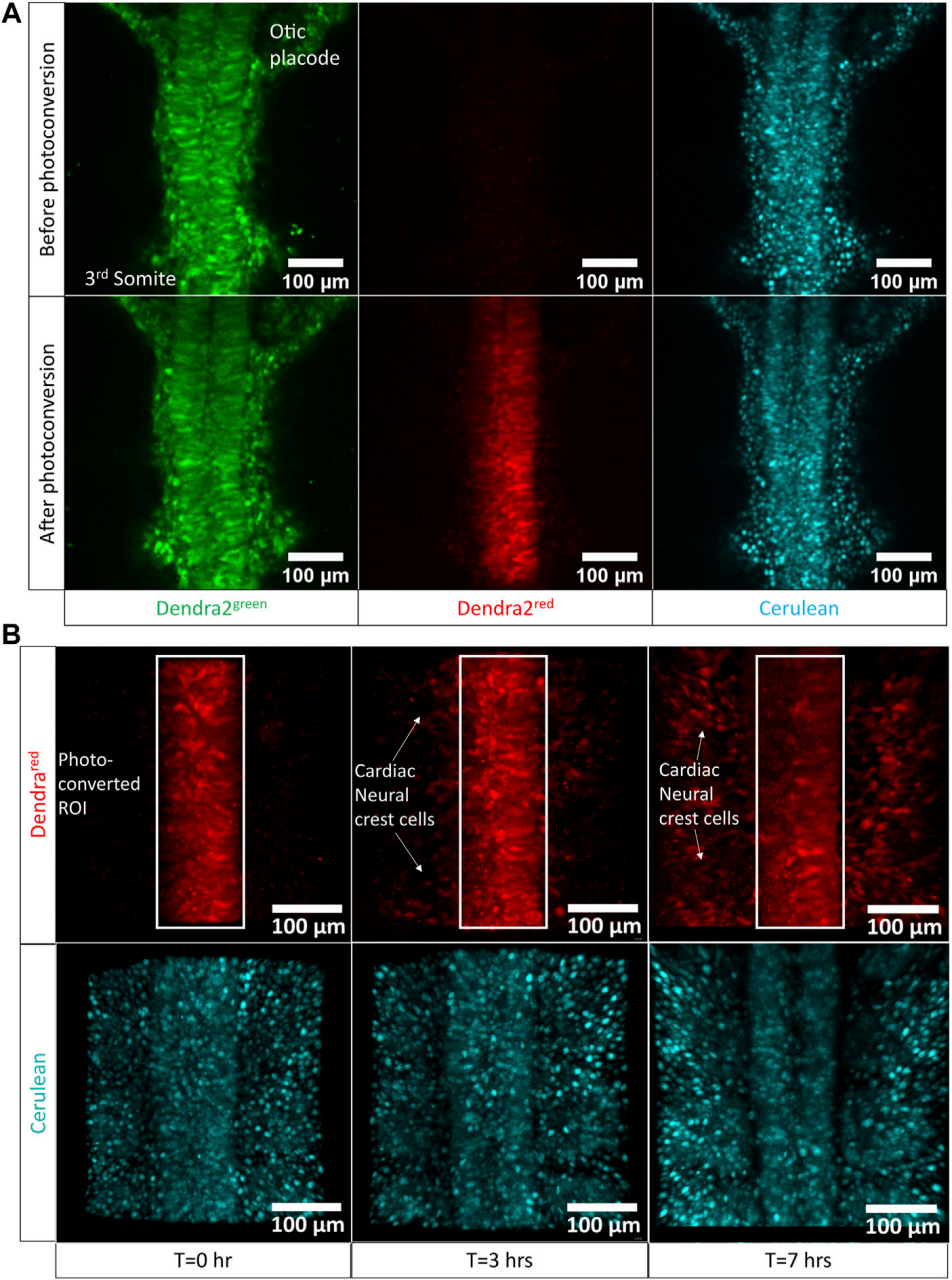
Cardiac neural crest cell migration imaged by photoconversion of an HH10 [Tg(hUbC:H2B-Cerulean-2A-Dendra2)] embryo using the egg-in-cube system. **(A)** Confocal images of the native green form of Dendra2 (referred to as Dendra2^green^ here), photoconverted red form of Dendra2, and Cerulean channels in an HH10 [Tg(hUbC:H2B-Cerulean-2A-Dendra2)] embryo before and after photoconversion (PC). The neural tube cells between the otic placode and the 3rd somite expressing the Dendra2^green^ are photoconverted using the 405 nm UV laser to induce the activated red form of Dendra2 (referred to as Dendra2^red^ here) expression. The Dendra2 green fluorescence intensity decreases with its photoconversion into its red form whereas the fluorescent intensity of the Cerulean channel remains unchanged. **(B)** Confocal images of the photoconverted red form of Dendra and Cerulean channels at different time points were sampled from the time-lapse data at 0 h, 3 h, and 7 h acquired using the upright confocal microscope. Photoconverted cardiac neural crest cells migrate laterally out of the neural tube towards the somites. The original time-lapse files can be seen in Supplementary Video S5.

To demonstrate the use of the egg-in-cube system for imaging cardiac neural crest migration in embryos atop yolk, we photoconverted Dendra2 expressing cells in the neural tube between the otic placode and the 3rd somite of an HH10 [Tg(hUbC:H2B-Cerulean-2A-Dendra2)] quail embryo mounted in the cube using the 405 nm laser. The 780LSM upright confocal microscope was used to photoconvert and image the migration of cardiac neural crest cells over time. Images were acquired before and after photoconversion to confirm the extent of the Dendra2 photoconversion in the region of interest. Representative images in (Figure 4A) show the photoconverted region of interest. Photoconverted cardiac neural crest cells start migrating out of the neural tube and are positioned laterally closer to the somites at 7 h into the time-lapse (Figure 4B), Supplementary Video S4). The orthogonal slice view aids in the visualization of the neural crest migration and calculating the distance covered by the photoconverted CNC cells on either side of the neural tube (Supplementary Figures S4A, B). When we quantified the number of photoconverted cells at t = 0 h and t = 7 h, the number of cells had increased 2.5 fold (334 cells in the neural tube at t = 0 h, 890 cells total in the neural tube + CNCs at t = 7 h, Supplementary Figure S4C). This increase in number represents a faithful transmission of photoconverted Dendra2 fluorescent protein through successive cell divisions between cells in the neural tube marking a subset of migrating CNCs. The egg-in-cube system enables us to image the migration of cardiac neural crest cells and it potentially can be used for *in vivo* manipulations of signaling pathways targeting this migratory behavior to further understand the role of these cells in embryo development.

### Imaging Cell Movements in the Early Blastoderm

We were also curious about how embryo development proceeds if we flipped the egg-in-cube so that cell movements could be imaged on inverted microscopes. In the case of an inverted confocal microscope, the gap between the embryo and the transparency membrane shrinks to the presence of a thin layer of albumen (∼100 μm) between the embryo and the membrane surface. When we tested the growth of the embryo in inverted cubes (*n* = 3 embryos in cubes) and incubated for 48 h, embryos developed marginally slower (by 3–4 h as compared to the expected stage), probably due to an unnatural yolk weight on the developing embryo (data not shown here). However, these embryos were morphologically normal for the period of development observed. In contrast, embryos from egg incubated controls developed normally.

**FIGURE 5:**
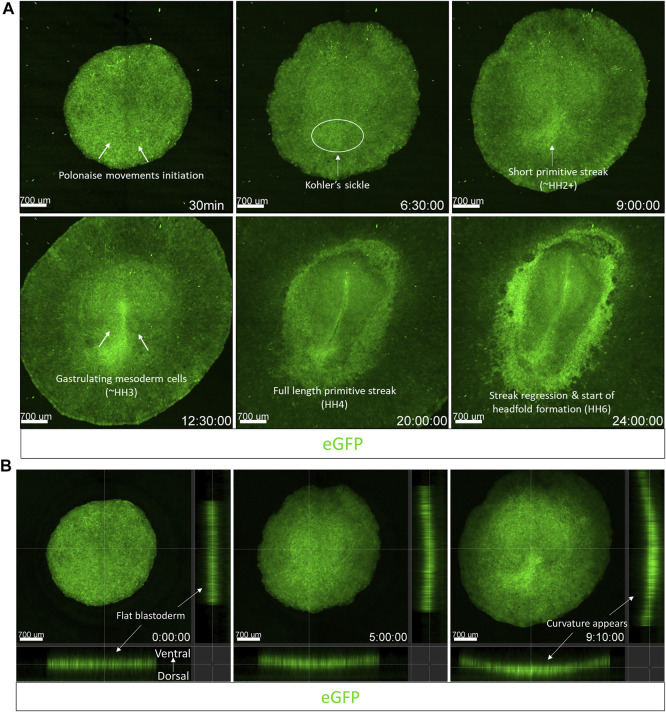
The egg-in-cube system enables dynamic imaging of early embryonic development. Captured frames from live confocal microscope imaging of an EGK-X [Tg(hUbC.membrane.EGFP)] quail embryo. The 5x images, with ×0.6 optical zoom, are maximum intensity projections of 11 optical slices (100 µm each) taken at 10 min intervals taken from the dorsal aspect of the embryo on the inverted confocal microscope. **(A)** Frames are shown at different time points highlighting key features of early avian development through the first 24 h of development. **(B)** Frames shown at different time points highlight the expansion of the blastodisc in 3D following its natural curvature on the yolk. Time-lapse images were acquired using the Zen 2011 (black) software, the acquisition was halted momentarily to readjust the focus and resumed several times. Acquired images were converted to a maximum intensity projection and stitched together in time to present the development as a continuous time-lapse. Images shown in **(B)** were taken from the first 9.1 h of development in 4D and presented as an orthogonal slice view using Imaris. The video files can be seen in Supplementary Video S5A (Panel 5A) and Supplementary Video S5B (Panel 5B).

Considering our observations from the embryo growth experiments above, we demonstrate the confocal time-lapse imaging of the first 24 h of avian embryo development using an EGK-X [Tg(hUbC.membrane.EGFP)] quail embryo in the egg-in-cube system. The embryo in the cube is inverted and placed in the on-stage incubator of a Zeiss LSM780 inverted confocal microscope for dynamic imaging. Cells in the lateral posterior edges of the EGK-X embryo start to move towards the midline to initiate the polonaise-like movements in the epiblast at 30 min (Figure 5, Supplementary Video S5A). These movements give rise to the prospective primitive streak as a small triangular structure at 9 h (HH2) and elongate to half its final length by 12 h into the time-lapse (∼HH3+). During these early events, the blastoderm expands not only in the X-Y dimension but also elongates in the Z dimension along its natural crescent-shaped curvature on the yolk, which also becomes apparent (Figure 5B, Supplementary Video S5B). We used the orthogonal section view in Imaris to quantify the change in the length and curvature of the blastoderm in the first ∼10 h of development. The blastoderm varies from being a flat disc-like shape at EGK-X: 3.3 mm length to a curved structure with an arc length of 6.5 mm at HH2+ (Supplementary Figure S5A). As gastrulation continues, prospective mesoderm cells can be seen arising in the form of mesodermal wings from either side of the elongating primitive streak (visible by 12.5 h HH3). By 20 h (HH4), the mesodermal wings have reached the anterior halves on both sides of the germinal crescent as the streak reaches its full length. Hensen’s node becomes apparent by 22 h, and the streak begins to regress as the head fold begins to appear by 24 h (HH6). The embryo gets brighter with development due to the accumulation of EGFP in the cell membranes. Using the egg-in-cube system, we report the imaging of the first 24 h of avian embryo development starting from EGK-X.

### Imaging the Migration of the Hypoblast Using Dendra2 Photoconversion

**FIGURE 6:**
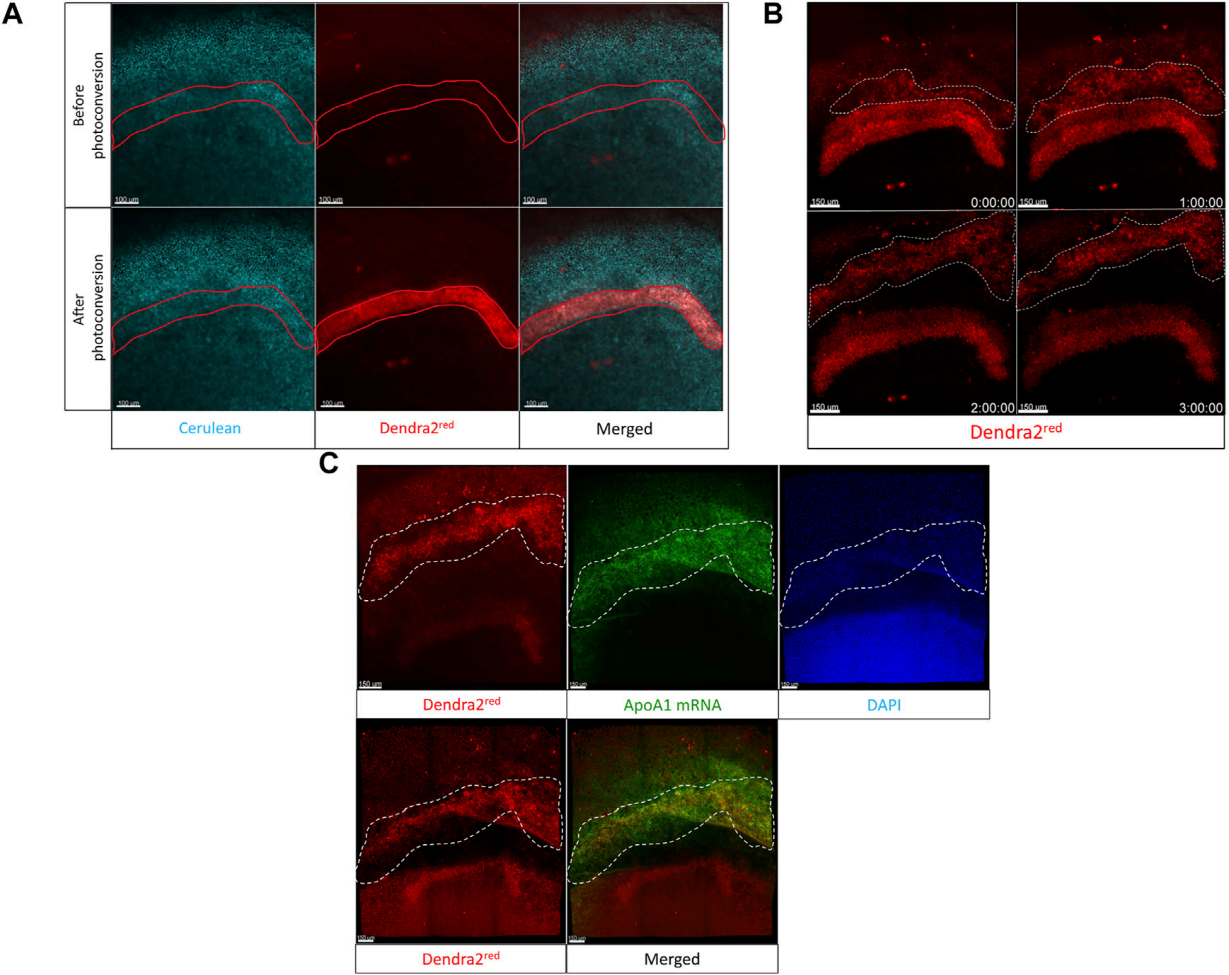
The Egg-in-cube system enables imaging of hypoblast migration. **(A)** Panels show the putative hypoblast region of interest in the anterior germinal crescent of an HH2 [Tg(hUbC:H2B-Cerulean-2A-Dendra2)] quail embryo before and after photoconversion. The 405 nm UV laser is used to photoconvert a region of interest using the “Regions” and “bleaching” function in Zen Black. Nuclei are labeled by Cerulean fluorescent protein (Cyan). Photoconverted cells are labeled by Dendra2^red^ fluorescent protein (red). Scale: 100 µm. **(B)** The migration of cells (red cells, region bounded by a white dotted line) in the photoconverted region of interest is shown over different time points (see Supplementary Video S6). Captured frames from live confocal microscope imaging from the dorsal side of an HH2 [Tg(hUbC:H2B-Cerulean-2A-Dendra2)] quail embryo. Images are maximum intensity projections of ×20 tiled images (24 slices of 6 µm) with ×0.6 optical zoom acquired every 10 min. Scale: 100 µm. **(C)** The last frame of the time-lapse (Dendra2^red^, upper left panel, 3 h) is compared to the Dendra2^red^ channel (Lower left panel) to demonstrate the position of the hypoblast layer (region bounded by a white dotted line in all panels) before and after ApoA1 HCR staining. Images are maximum intensity projections of ×20 confocal Z stacks (20 slices of 8 mm) from whole-mount *in situ* hybridization for hypoblast specific ApoA1 (green) staining within an HH2 [Tg(hUbC:H2B-Cerulean-2A-Dendra2)]. DAPI stain in the nuclei is shown as blue. Scale: 150 µm.

To image the anterior migration of the hypoblast, we used stage HH2 (10 h of incubation) embryos from the [Tg(hUbC:H2B-Cerulean-2A-Dendra2)] quail line and transferred multiple embryos to cubes. Embryos were screened for the brightest Cerulean-Dendra2 fluorescence, and the selected embryo was then transferred to the stage of the inverted confocal microscope. We photoconverted cells (Huss et al., 2019) in an arc-shaped region of interest (ROI) in the putative migrating hypoblast front (Figure 6A). This photoconverted cell layer was then tracked over time. The time-lapse analysis showed that a group of cells migrating like a collective sheet separated from this photoconverted ROI (white dotted selection, Figure 6B, 1 h–3 h, Supplementary Video S6) and migrated towards the anterior germinal crescent of the embryo. An *in situ* hybridization staining with probes against ApoA1 mRNA immediately following the time-lapse, allowed us to validate the identity of the tracked cells as hypoblast cells from the photoconverted region (white arrows, Figure 6C, top panel, left-right). Using the egg-in-cube system along with the photoconvertible [Tg(hUbC:H2B-Cerulean-2A-Dendra2)] quail embryos, we were able to track the anterior migration of hypoblast cells.

### Electroporation of the Quail Neural Tube “in Cubo”

**FIGURE 7:**
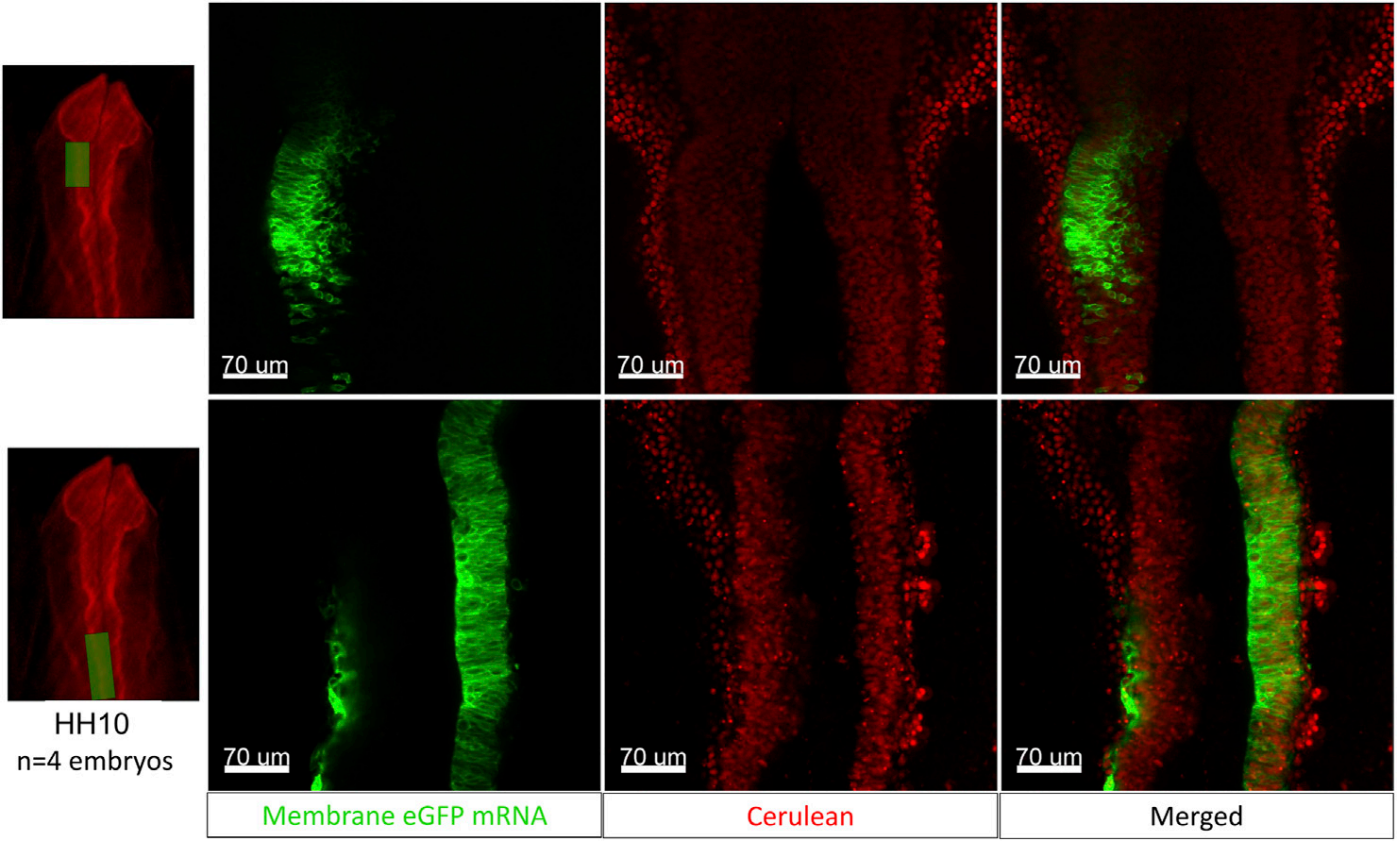
The egg-in-cube system enables electroporation of the neural tube with fluorescent mRNA. The images in the top panel show a representative midbrain region of the [Tg(PGK1-H2B-mCherry)] embryo (red nuclei) electroporated with membrane-eGFP mRNA (green) and the bottom panel shows a region caudal to the midbrain (closer to the hindbrain and otic placode). These images show a small proportion of H2B-mCherry expressing cells colocalizing with membrane eGFP post electroporation. Scale: 70 µm.

To demonstrate the increased accessibility of the developing embryo in the cube, we electroporate mRNA encoding membrane-GFP into an HH10-11 [Tg(PGK1:H2B-mCherry)] embryo unilaterally into the dorsal neural tube/midbrain-hindbrain region and image the resulting electroporation after 2 h of incubation at 37°C. Images from two embryos are presented in Figure 7, the top panel shows the left side of the midbrain region of one of the H2B-mCherry embryos (red nuclei) electroporated with membrane eGFP mRNA (green) and the bottom panel shows a region caudal to the midbrain from the second electroporated embryo (closer to the hindbrain and otic placode).

### Optimizing the Use of the Cube on Different Microscopes

We observed that quick lateral movements cause the embryo to slosh about both in cubo and in ovo. This is not unexpected since the yolk is surrounded by thick and thin liquid albumen layers. Embryo displacement can cause serious image registration issues if the movements occur during time-lapse imaging that might prevent accurate post hoc cell tracking and analysis. For instance, motorized stages are often used during time-lapse imaging experiments to cover and collect larger regions of interest (ROI) with high spatial resolution. Indeed we noticed during post hoc analysis that stage movements on some microscopes induced embryo displacements that we did not notice by eye during the experiments.

The stage speed on our Zeiss 780LSM inverted confocal microscope in the lab was seen to be slower than the Zeiss 780 LSM upright version at a different location. This slower stage speed helped to prevent the sloshing of the yolk in the X-Y direction and leads to seamless stitching between tiles after the time-lapse images have been acquired. To circumvent this problem on other microscopes, we came up with the heat fixation method for stabilizing the yolk. Using a hot soldering iron, we can denature the yolk in contact with the PDMS membranes to bind it to the four sides of the cube and make it more resistant to sloshing even at faster stage speeds.

Another essential consideration for the choice of imaging modality for any egg-in-cube experiment is the developmental stage of the embryo. The embryo in the cube is more stable and resistant to yolk rotation in the first 24 h of development, and hence either an inverted or upright microscope can be used for imaging in these stages. As the embryo grows in 3D and becomes heavier, yolk rotation becomes a significant factor, and an upright imaging microscope is better suited for imaging post 24 h of incubation.

It is well-known that egg composition can vary in weight significantly with different factors like breed, flock age, strain, and even within the flock breed (Friese, 1923; Scott & Warren, 1941; Tolman & Yao, 1960; Fletcher et al., 1981; Hussein et al., 1993). This variability in egg component weight, specifically the size of egg yolk causes some of the embryos with a smaller yolk volume to lose contact with one or more surfaces of the cube when mounting the embryo into the cube for imaging and makes it prone to sloshing as described above. To deal with this problem, we added sterilized glass beads into the cube after adding the egg contents to raise the egg yolk and maintain the embryo close to the imaging surface of the cube. Another method that can be applied to raise the height of the embryo, is to add ∼1 ml of molten bacto agar before adding the yolk. This bacto agar bed solidifies and essentially reduces the height of the cube temporarily and can be used for eggs with smaller yolk sizes. Another advantage of the bacto-agar method is the flexibility of decreasing the available cube height as desired by the user. Both the sterilized beads and the bacto agar bed, marginally flatten the yolk from its equator, increasing contact with the cube surface and allowing heat fixation for smaller yolks to provide better stability in case of inverted confocal imaging.

## Critical Observations From the Egg-In-Cube System

We have engineered an egg-in-cube system that permits embryogenesis to be dynamically imaged in its native state atop the yolk. In the experiments shown here, we have used the quail egg-in-cube system to explore different developmental events occurring in avian embryos using different imaging platforms. The egg-in-cube system provides easy access to the developing embryo to carry out a variety of experiments, including tissue transplantation, microinjection, a viral infection of early embryos, electroporation of DNA/RNA or morpholinos into the embryos, and setup dynamic imaging in a short interval of time.

We first imaged the development of the quail embryo from EGK-X until 11.25 days of incubation using the egg-in-cube system, which corresponded to a developmental stage of HH35 (∼E8) for the embryo. This is the first report of an ex ovo culture system being able to image the first 8 days of quail embryo development. Several studies in the literature report the effects of hyperoxia on avian embryos at different levels of O_2_ supply above the normoxic condition (ranging from 25 to 100% O_2_) (Stock and metcalfe, 1984; Metcalfe et al., 1981; Höper and Jahn, 1995; Lourens et al., 2007). Experiments by Stock and Metcalfe. (1984) have described an accelerated embryo growth with chick embryo incubation at 60% O_2_ compared to controls incubated in the air (20.9% O_2_). We reasoned that we might be able to decrease the lag in embryo development and improve the embryo survival rates observed in the egg-in-cube system using the 60% O_2_ condition. Kaplan Meier’s Survival curve analysis showed that the embryos incubated in 60% O_2_ had a significantly higher chance of surviving longer with a median survival time of 14 days. This system, in its current form, can achieve a 50% embryo survival rate up to 14 days of incubation in the cube initiated from EGK-X (8/15 embryos, Figure 1B) at 60% O_2_ with minimal interference to the system during the culture period. The lag in embryo growth when compared to an in ovo control also reduces from ∼3 days (Air incubation, 20.9% O_2_, Figure 1A) to ∼24 h (60% O_2_, Supplementary Figure S6A). Additional characterization of the embryo culture conditions with a hyperoxic incubation environment will be needed to further minimize the embryo development lag and enable the hatching of embryos using the egg-in-cube system. This system proves to be a powerful tool to understand the development of avian embryos through long-term culture and longitudinal imaging.

We demonstrate that the egg-in-cube system with a custom incubator can be used for embryo culture and dynamic imaging of the fluorescent quail embryo starting from the egg-laying stage (EGK-X). Though a modified ex ovo New culture has been successfully used to image the development of the chick embryo from EGK-XII (2 h of incubation) (Voiculescu et al., 2007), the cube system makes it possible to study the cell movements in the freshly laid avian blastoderm. Also, when the cube is used in an upright imaging modality, we can recapitulate the proper tissue tension and mechanical forces that occur in the embryo during its development in the egg.

During the first few hours of incubation, the primary hypoblast arises from the upper epiblast layer by the process of polyingression (Vakaet, 1962, 1970). This primary hypoblast is carried by the anterior migrating endoblast and spreads anterio-laterally, giving rise to the developing extra-embryonic (yolk sac) endoderm (Eyal-Giladi and Kochav, 1976; Sanders et al., 1978). One of the disadvantages of the ex ovo culture system for imaging development of the hypoblast is the difficulty in maintaining the lower layer (hypoblast) of cells intact while isolating the embryo at stages earlier than HH2. Using the fluorescent transgenic [Tg(hUbC:H2B-Cerulean-2A-Dendra2)] quail line in the egg-in-cube system, we photoconvert and track the anterior migration of the putative hypoblast layer. The time-lapse is immediately followed by *in situ* hybridization staining against a specific hypoblast marker ApoA1 to confirm the identity of the tracked cells as the hypoblast. The egg-in-cube system along with photoconvertible fluorescent transgenic quail embryos opens up the avenues to better understand the process of hypoblast polyingression and migration dynamically in living embryos.

Along with the formation of the hypoblast, primordial germ cells are specified in the epiblast layer and also undergo delamination to meet the anterior migrating hypoblast and reach the extra-embryonic space by HH4 (Swift, 1914; Eyal-Giladi et al., 1981). The specification of primordial germ cells has only been studied using static snapshots over development. With the combined power of the egg-in-cube system and fluorescent transgenic quail, it will become easier to image these different developmental events using multiphoton microscopy, which would be technically challenging to accomplish by ex ovo culture.

The in ovo electroporation technique for chick embryos has been developed as an essential method to introduce plasmids/viral vectors for gain or loss of function studies in development (Muramatsu et al., 1997; Funahashi et al., 1999). In this work, we demonstrate the electroporation of the quail neural tube with *in vitro* transcribed membrane-GFP mRNA followed by a short 2-hour embryo incubation and imaging of the transfected embryo. The egg-in-cube system may also be useful for dynamic imaging of fluorescent protein maturation, and translocation into targeted organelles post electroporation *in vivo*. In our previous study (Tran et al., 2019), we have shown that when we electroporate ex ovo cultured HH5 quail embryos, mRNA encoded fluorescent proteins (FPs) are expressed within 22 min of electroporation with 75% efficiency as compared to DNA encoded FPs (∼3–6 h post electroporation with 25% efficiency). This technique of “in cubo” electroporation makes it easier to transfect cells in the avian embryo with mRNA encoded fluorescent proteins, dominant-negative expressing constructs & introduce knockdown reagents, followed by “in cubo” culture. The egg-in-cube system will enable dynamic/static microscopy of the transfected embryos to observe the phenotype caused by the perturbation.

In the first 3 days of incubation, the avian embryos can easily be manipulated by a “window” in the eggshell and re-incubated until the desired stage for observation. This property of avian embryos has led to the development of several classical techniques for surgical manipulations, tracing cell migration by DiI injections (Kulesa & Fraser, 2000), tissue transplantation/ablation studies (Le Douarin, 1973; Goldstein, 2006; Lwigale & Schneider, 2008) for studying morphogenesis and cell fate. The egg-in-cube system makes the embryo accessible to all these manipulation techniques and enables the direct observation of its effects on the embryo through static/dynamic imaging. We have shown that the cube is easily amenable to the insertion of surgical tools and manipulation of blood vessels and tissues in the chick system. We have also used the cube as a platform to study angiogenesis and induction of blood vessels in the chick CAM (Huang et al., 2017). This system will also make it convenient to study the function of lipid uptake/metabolism by the introduction of fluorescent lipid tracers or chemical inhibitors through yolk injection in the cube. One of the few disadvantages of this culture system for dynamic imaging is the inability to directly access the ventral side of the avian embryo as it remains in yolk for the first few days of development.

In this study, we have demonstrated the use of the egg-in-cube platform using the simple phone camera, an upright fluorescent stereoscope, and the upright/inverted confocal microscopes for imaging quail embryo development. The cube can potentially also be used on any other imaging platform in the upright/inverted modalities like the light sheet microscope, with minor stage modifications. We have adapted the egg-in-cube system developed for the chick embryo in Huang et al. (2015) to the quail embryo here. The concept of the cube can be extended easily to other avian species to empower research in these model systems, potentially making it valuable to other oviparous model systems too.

## Data Availability

The original contributions presented in the study are included in the article/Supplementary Material, further inquiries can be directed to the corresponding authors.
