# Supplementary material for: A Novel Egg-In-Cube System Enables Long-Term Culture and Dynamic Imaging of Early Embryonic Development
Source: Front Physiol. 2022 May 12;13:893736. doi: 10.3389/fphys.2022.893736 (PMC9133561; doi:10.3389/fphys.2022.893736)
Supplement: Supplementary file 2 [file DataSheet1.PDF]

Supplementary Material

1.1 Supplementary Figures

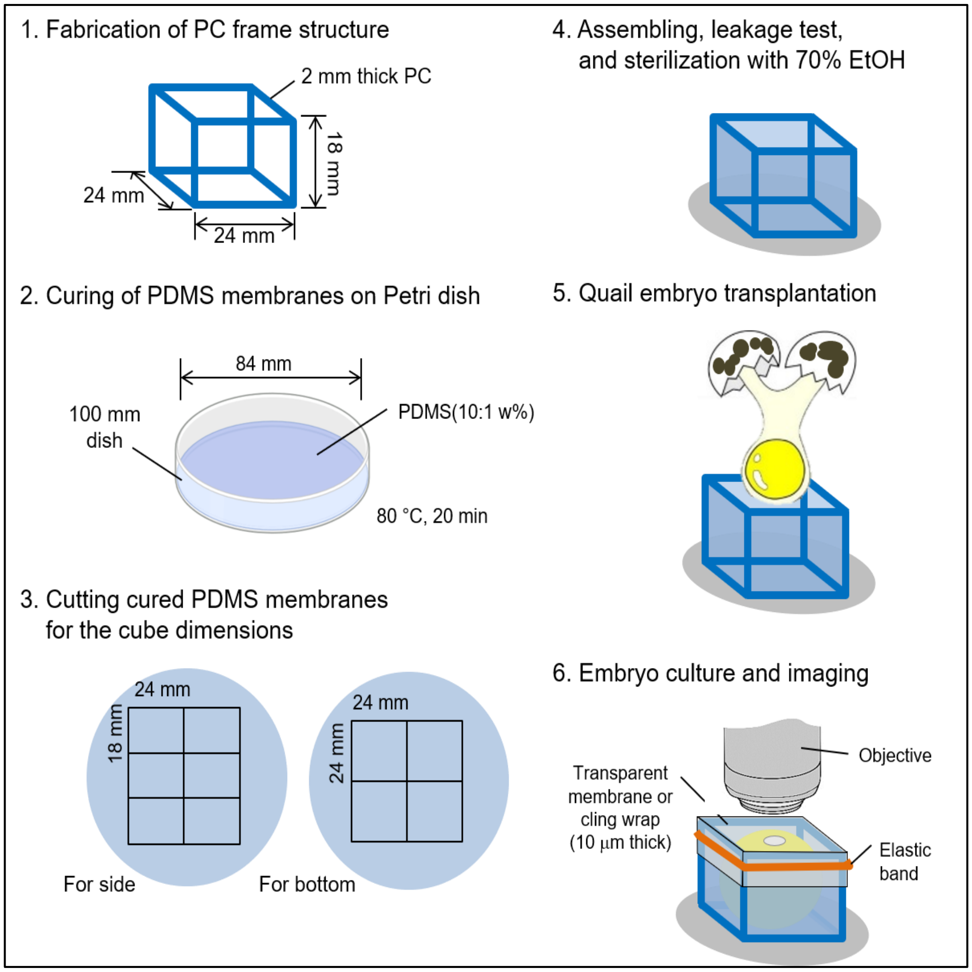

Supplementary Figure 1: Fabrication process of the quail egg-in-cube system: 1) A hollow cubic frame was manufactured using a polycarbonate (PC) material (PCP1609A, Takiron Co., Ltd.) by machining (α-T14iF, FANUC Co., Ltd.). **2)** PDMS membranes with 300µm thickness were fabricated on the surface of a petri dish glass substrate by using a mixture of PDMS (Sylgard 184 Silicone Elastomer Base, Dow Corning) and a curing agent (Sylgard 184, Dow Corning) at a 10:1 ratio, and then cured on a hotplate (80°C, 20 min). **3)** The cured membrane was stretched out on a sterile surface and cut into the dimensions of the sides of the cube (L*B*H: 24*24*18mm). **4)** PDMS membranes were then attached to the hollow frame using PDMS glue and cured on the hotplate (80°C, 20 min), with the top surface unwrapped. The cubic eggshell was filled with distilled water to confirm the lack of leakage. The eggshell was then sterilized by distilled water and 70% Ethanol before transferring the egg contents. **5)** The contents of a fertilized quail egg were transferred into the cubic eggshell. **6)** A rectangular piece of high transparency membrane (High sensitivity stretch membrane, YSI/Xylem Inc., # 098095) or cling wrap was stretched taut on top of the open cube surface. An elastic rubber band was stretched around the cube to stabilize the membrane and seal the cube.

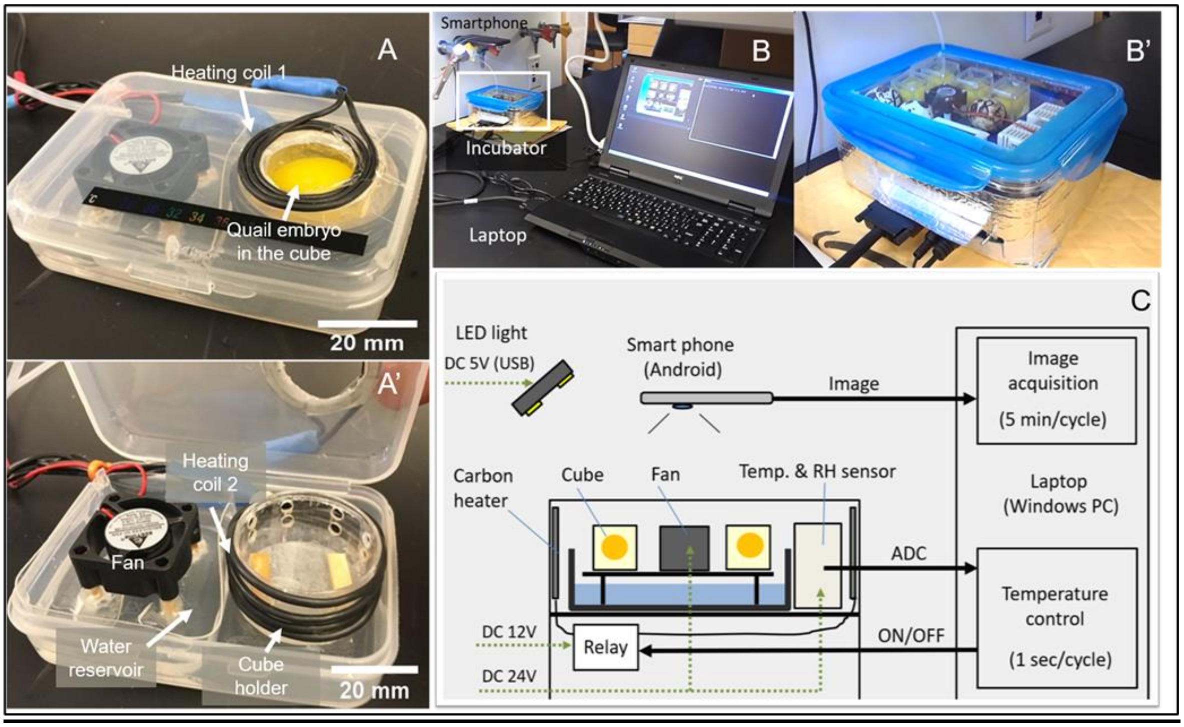

Supplementary Figure 2: Customized incubators built for the quail egg-in-cube system. A-A') For onstage incubation: The customized box incubator is a plastic box equipped with a cube holder encased by heating coils around its side and top for providing a constant temperature of 38°C. The water reservoir helps maintain humidity in the incubator. A portable temperature and humidity sensor can be inserted into the box to measure the environmental conditions close to the edge of the cube. This setup is adaptable to both inverted and upright microscopes. **B)** Imaging setup for long term embryo culture and imaging: Contents of fertilized quail eggs were transferred to several cubes and transferred to the incubator along with intact eggs as controls. An Android phone was setup just above the incubator, it was connected to a laptop and programmed to acquire images at regular intervals. A USB LED light was used to maintain a constant field of bright light on the incubator surface. **B')** A zoomed in image of the incubator shown in B). **C)** A block diagram explaining the system components (in B-B') involved for the brightfield imaging of embryos from EGK-X stage. Image acquisition from the smartphone camera is controlled by the laptop at 5 min intervals.

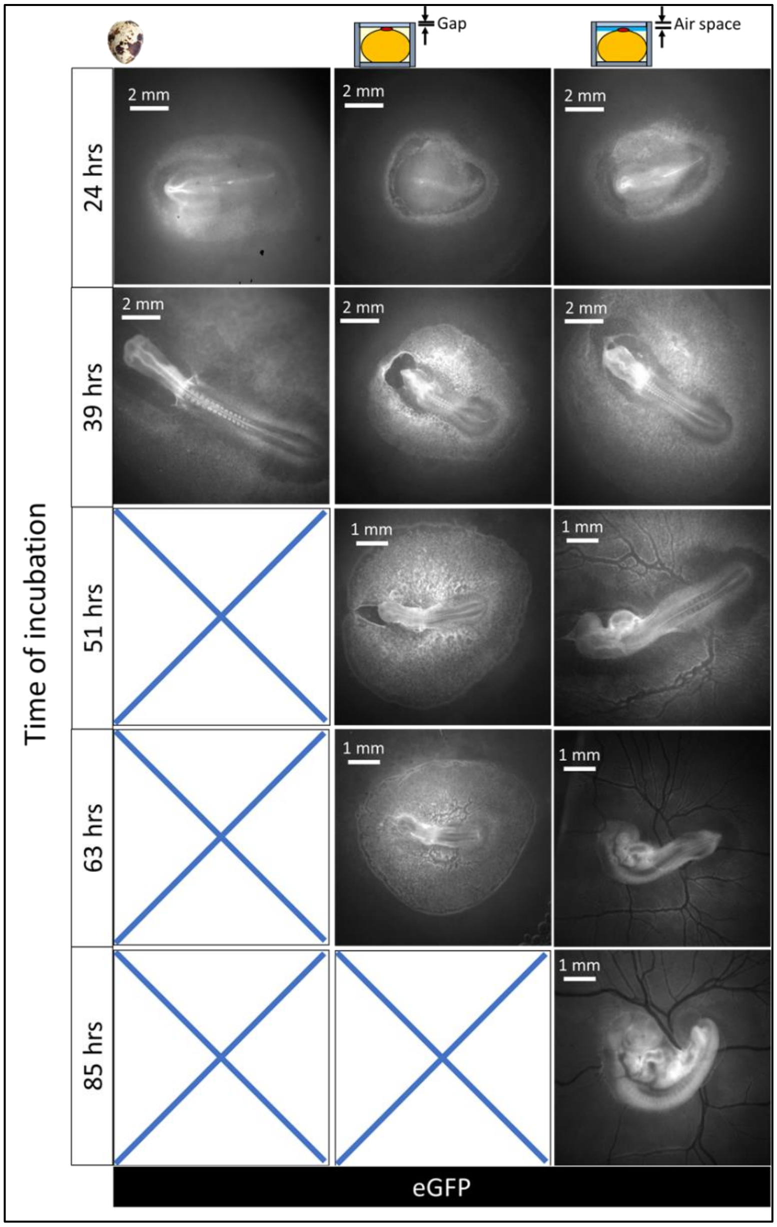

Supplementary Figure 3: Embryo development in cubo improves with a small air gap above the embryo: Captured images at different time points for EGK-X [Tg(hUBC.membrane.EGFP)] quail embryos cultured in three conditions: In the egg, in the cube with no air gap and with an air gap over 85 hours of incubation. "X" marks the time point at which the embryo was harvested from the egg (egg incubation) or the embryo died (with no air gap incubation). The embryo in the cube with a small air gap develops normally like that in the egg.

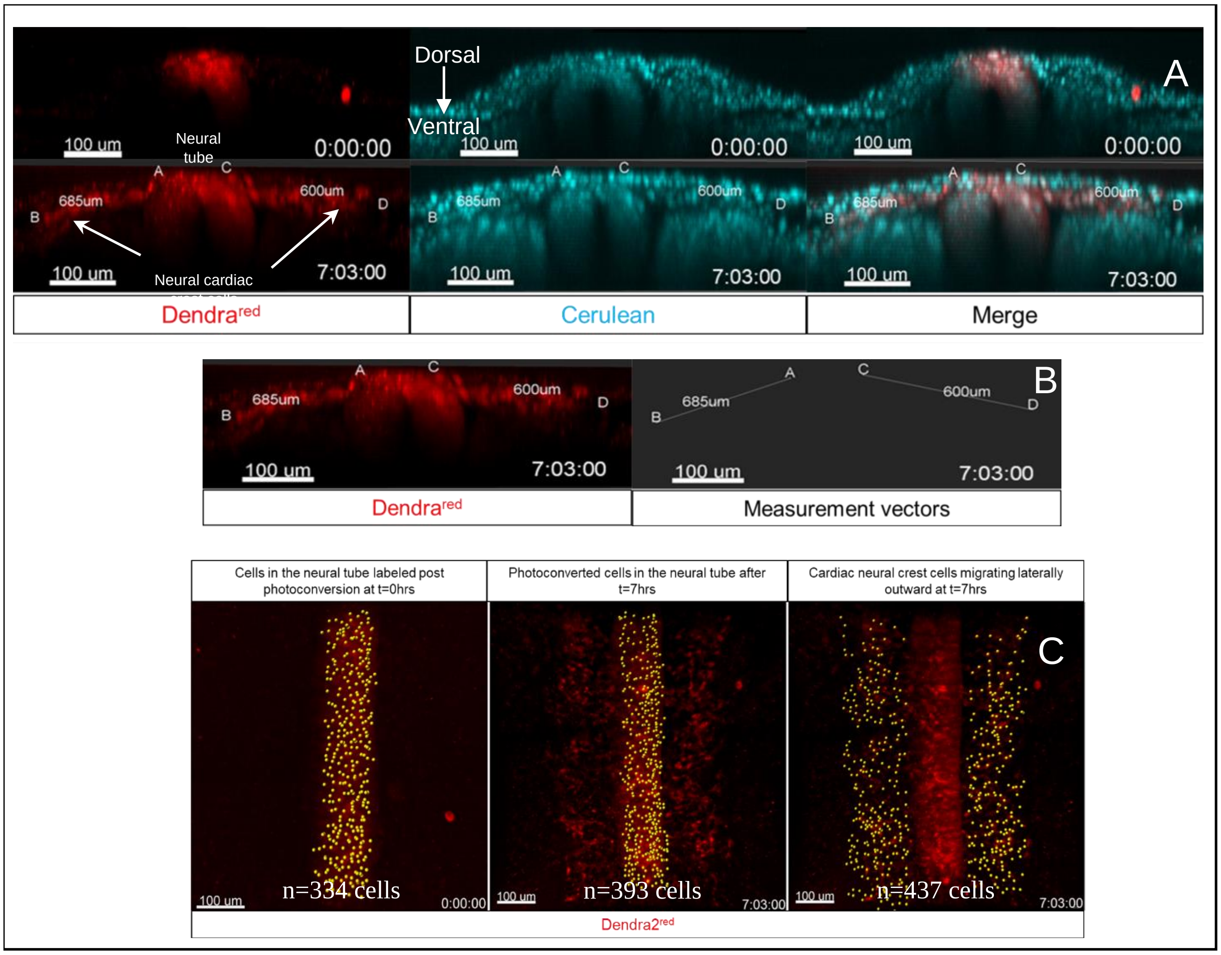

Supplementary Figure 4: Analyzing cardiac neural crest cell migration using Dendra2 photoconversion.

A) The figure panel shows the position of the photoconverted cells at t=0 hrs and t=7:03 hrs. The orthogonal slicer tool in Imaris 9.5 was used to visualize the X-Z section view of the neural tube. The width of the section visualized:100µm and the section visible in the figure panel is around the 1st somite pair. Cells in the section are labelled as Dendra2^{red}(red), nuclei are labeled as Cerulean (cyan) are also shown here. **B)** The measurement tool in Imaris was used to calculate the distance travelled by the neural crest cells from the edge of the neural tube at t=7:03 hrs. **C)** The Spots tool in Imaris was used to quantify the number of cells photoconverted at t=0 hrs, number of photoconverted cells in the neural tube and migrated laterally towards the somites at t=7:03 hrs shown as yellow spheres.

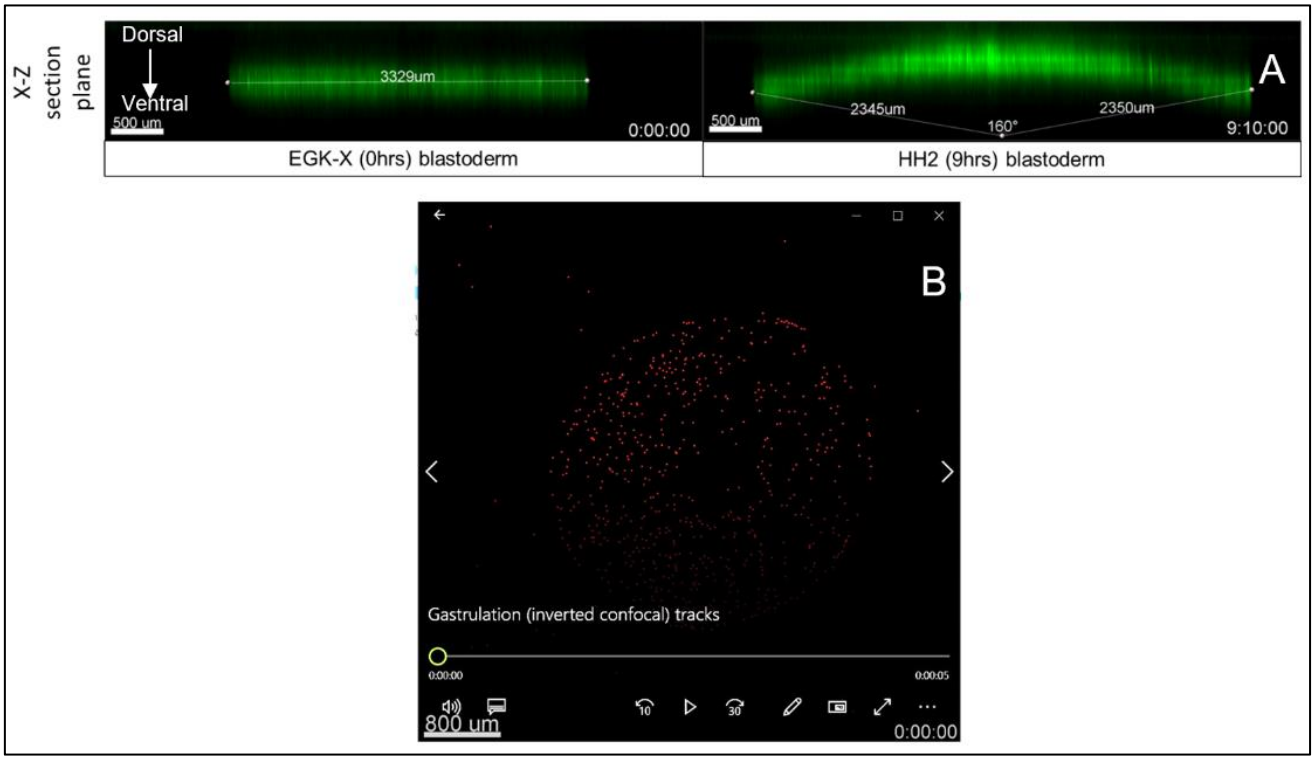

Supplementary Figure 5: Analysis of the tissue movements during gastrulation in a Tg(hUBC.membrane.eGFP) quail embryo in the cube from EGK-X. A) The time lapse data obtained on the inverted confocal microscope was analyzed using the orthogonal slicer tool using Imaris 9.5. The figure panel here shows the Y-Z section view of the embryo at t=0 hrs and t=9 hrs. The measurement tool in Imaris was used to calculate the width and arc radius of the embryo. **B)** Imaris was used to perform spots and cell tracking on the time lapse data (acquired in **Supplementary Movie 5B**) to analyze tissue movements seen during the early phases of primitive streak formation (red spots with yellow tracks, shown as **Supplementary Movie 5B**).

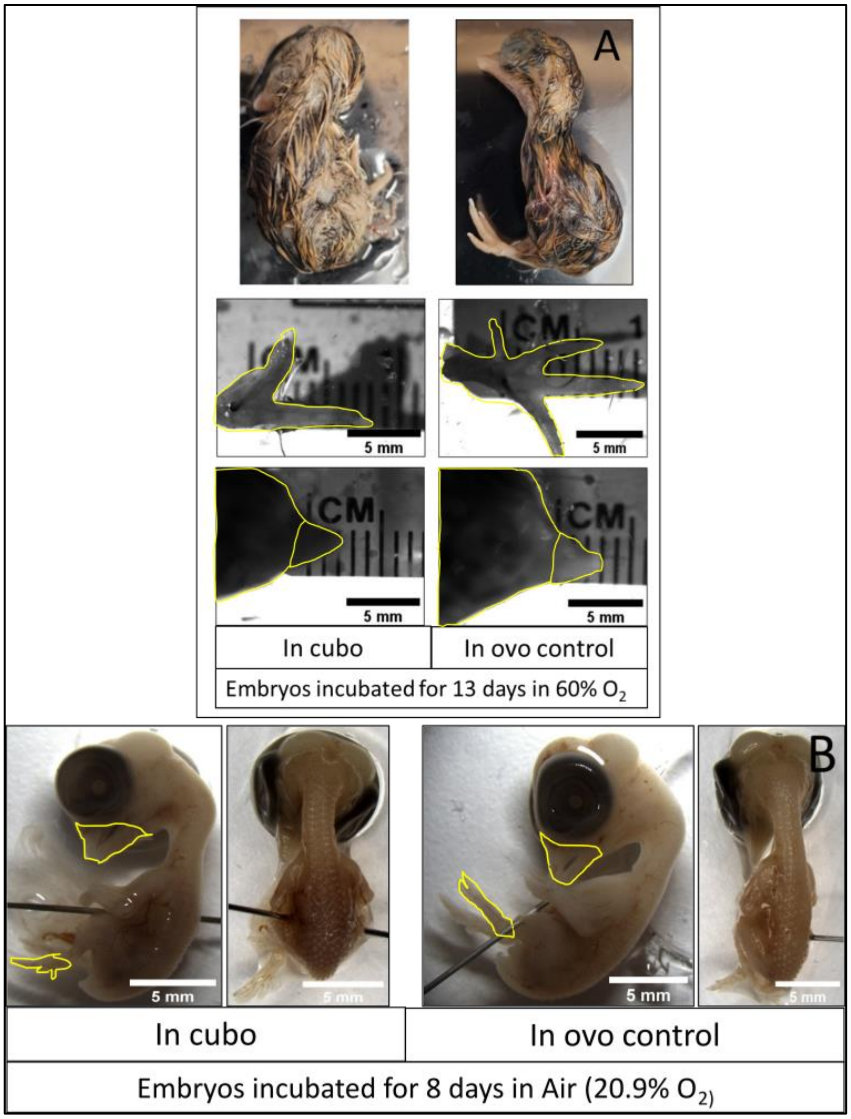

Supplementary Figure 6: Comparative morphological analysis of embryos incubated in the cube at 60% O_2 versus air (20.9% O_2). Representative images of wild type embryos incubated in the cube either at 60% O_2 condition **A)** or in Air (20.9 % O_2) **B)** shown along with their respective experimental in ovo controls. The panels show an overall view of the embryos and outline the staging method used in this study. The length of the third toe and beak (yellow outlines) were measured for all embryos incubated and the staging was done as per morphological features described in Ainsworth et al., (2010) and Hamburger and Hamilton (1951). **A)** The embryos shown here lived up to 13 days of incubation in 60% O_2, after which the embryo was fixed and staged with an embryonic age of HH42 or E12-13. The corresponding in ovo control was staged at an embryonic age of HH43 or E13. **B)** The embryos shown here lived up to 8 days of incubation in Air (20.9% O_2), after which the embryo was fixed and staged with an embryonic age of HH35 or E8. The corresponding in ovo control was staged at an embryonic age of HH36 or E8.

1.2 Supplementary movies legends

Supplementary Movie 1. Imaging embryo development over 11days of culture in the egg-in-cube system. Time-lapse imaging of a wild type quail embryo transferred to the cube at EGK-X. Embryos in 6 different cubes were placed for long term culture and imaging in the big box incubator shown in Fig S2B. The embryo with the longest developmental time is shown here as an example. The embryo is oriented with its anterior end on the top. Through the first day of incubation, the embryo expands with the edges of the blastoderm, covering almost all the visible yolk boundaries. Into day 2, the vasculature develops, and the heart starts to beat by ~48 hrs of incubation. By day 3-4, the embryo is more visible with its expanding yolk sac vasculature. The allantoic vesicle develops from the start of day 5 in the movie. The embryo starts growing rapidly in size with its head, eyes, and limbs becoming bigger from day 6. The blood vessels in the developing chorioallantoic membrane cover the embryo and the walls of the cube. The yolk sac vasculature expands and covers the yolk as the embryo grows bigger. The time-lapse ends by 11days 14 hrs of incubation from EGK-X, when the yolk sac vasculature begins to lose its red color, and the embryo dies. Dorsal view. Scale:5 mm

Supplementary Movie 2. Imaging development of the embryo from EGK-X using an upright fluorescent stereoscope. 2D rendering of embryonic cells labeled with membrane-localized EGFP and imaged by time-lapse fluorescence microscopy over 60 timepoints at 10-min intervals for a total of 9:40 hrs. For the time-lapse, a Tg(hUBC.membrane.eGFP) quail embryo transferred to the cube at EGK-X. This embryo was mounted into the cube in the custom box incubator shown in Fig S2A. and set on the stage of the upright Olympus fluorescent stereoscope for dynamic imaging. The embryo is oriented with its anterior end on the top. The cells in the posterior edges start moving towards the center in the "polonaise" fashion by 1hour of incubation. By ~3:20, a short primitive streak develops, which is accompanied by anterior migration of the presumptive hypoblast cells. By the end of the movie at 9:40, the primitive streak has reached half its maximum length, and the hypoblast cells (now visible as a crescent-shaped layer) have migrated closer to the anterior edge of the embryo. The embryo becomes noticeably brighter as it develops. Dorsal view. Scale:700 μm

Supplementary Movie 3. Using fluorescent bead injections to study blood flow dynamics in embryos using the cube on an upright fluorescent stereoscope. 2D rendering of far-red fluorescent beads injected into extraembryonic circulation and imaged by time-lapse fluorescence microscopy over 152 timepoints at 50ms intervals for a total of 7:06sec. For the time-lapse, a wild type E3 quail embryo was transferred to the cube. This embryo was mounted into the cube in the custom box incubator shown in Fig S2A. and set on the stage of the upright Olympus fluorescent stereoscope for dynamic imaging. The anterior region of the embryo is oriented to the left of the screen. **A)** The region shown in the movie A is close to the left vitelline vein in the extraembryonic vasculature. 9s into the video, a rectangular box marks the region of interest zoomed into in movie B. **B)** Video B is modified from Video A; the image has been cropped in the *xy* dimensions and later in the time '*t*' to trace the movement of a fluorescent bead through the smaller capillaries. The bead being traced is marked with a circle as it moves through smaller capillaries and eventually joins a larger vessel connecting to the vitelline vein. This movie is used to find the speed of the blood circulation by tracing the displacement of the fluorescent bead through the frames observed. Dorsal view. Scale:150 μm in A and 50 μm in B.

Supplementary Movie 4. Multispectral 4D imaging of cardiac neural cell migration using the cube on an upright confocal microscope. 4D rendering of neural tube cells labeled with cytoplasmic Dendra2, photoconverted using the 405nm laser and imaged by confocal time-lapse microscopy over 53 timepoints at 8 min intervals for a total of 7:03 hrs. For the time-lapse, a Tg(hUbC:H2B-Cerulean-2A-Dendra2) quail embryo transferred to the cube at HH10 (E2.5). This embryo was mounted into the

cube in the custom box incubator shown in Fig S2A. and set on the stage of the upright 780LSM confocal microscope. Cells in the neural tube between the otic placode and the third somite were photoconverted using the bleach function in Zen black with a 405nm laser line. Time-lapse images were acquired for the photoconverted Dendra2^{red}(red) and Cerulean (cyan) fluorescent proteins expressed in cells. The embryo is oriented with its anterior end at the top of the screen. The region of the neural tube photoconverted is shown with a rectangular box in the video shown here. Dendra2^{red} expressing cardiac neural crest cells start migrating bilaterally out of the neural tube by ~3 hrs of incubation. These cells steadily migrate laterally along their destined paths towards the developing pharyngeal arch arteries until the video is stopped at ~7 hrs. The embryo moves out of focus during imaging, and thus imaging is halted and readjusted multiple times. The video shown here is a concatenated series of original videos acquired. Dorsal view. Scale:130 µm in all videos.

Supplementary Movie 5. 4D imaging of tissue movements during the first 24 hrs of embryo development imaged in the cube on an inverted confocal microscope. 4D rendering of embryonic cells labeled with membrane-localized EGFP and imaged by confocal time-lapse microscopy over 149 timepoints at 10 min intervals for a total of 24:40 hrs. For the time-lapse, a Tg(hUBC.membrane.eGFP) quail embryo was transferred to the cube at EGK-X. This embryo was mounted into the cube, a 35 mm imaging dish was placed on top of the cube, and it was set on the stage of the inverted 780LSM confocal microscope. This microscope is equipped with an on-stage incubator to maintain the embryo at 38°C. A) The unincubated embryo grows with time, and the Koller's sickle is visible as a thickening in the posterior region of the embryo (~6 hrs). At HH2 (~9 hrs), a short triangular streak appears from the posterior end of the embryo, which elongates through HH3 (~13 hrs). As gastrulation occurs, cells start moving bilaterally from the primitive streak in the form of mesodermal wings (~13:30 hrs). These cells converge at the anterior end of the embryo by HH4 as the primitive streak reaches its full length, and the germinal crescent soon forms (~18:30 hrs). The top half of the embryo elongates as the Hensen's node starts to regress slowly by ~22:40 hrs until the end of the video at ~24:20 hrs. B) Video B has been modified from the A) using the section view on Imaris 9.5 during the first 9 hrs of development. The unincubated blastoderm starts as a thin planar disc at the start of the video. As the embryo grows in the X-Y direction through the first four hours, the blastoderm edge expands (as seen in all three section views), and the polonaise-like movements begin by ~4:00 hrs. With these polonaise movements and hypoblast migration, the embryo grows in 3D and gains its natural crescent-shaped curvature on the yolk (~5:30 hrs onwards). The movie has been halted and focus has been readjusted several times to keep the embryo within the plane of imaging. The video shown in A is a concatenated maximum intensity projection series of five-time lapse files. These post-imaging modifications were done in Zen 2011 (Black) after saving the original LSM files. The movie projection series of five-time lapse files. These post-imaging modifications were done in Zen 2011 (Black) after saving the original LSM files. The movie in B) is obtained using the section view function on Imaris 9.5 on the first time-lapse file (first 9 hrs of incubation). Dorsal view. Scale:130 µm in all videos.

Supplementary Movie 6 4D imaging of hypoblast migration using Dendra2 photoconversion. 4D rendering of putative hypoblast cells labeled with cytoplasmic Dendra2, photoconverted using the 405nm laser and imaged by confocal time-lapse microscopy over 53 timepoints at 8 min intervals for a total of 7:03 hrs. For the time-lapse, a Tg(hUbC:H2B-Cerulean-2A-Dendra2) quail embryo was transferred to the cube at HH2. This embryo was mounted into the cube in the custom box incubator shown in Fig S2A. and set on the stage of the upright 780LSM confocal microscope. Cells in the anterior migrating edge of the hypoblast were photoconverted using the bleach function in Zen black with a 405nm laser line. Time-lapse images were acquired for the photoconverted Dendra2red(red) and Cerulean (cyan) fluorescent proteins expressed in cells. The embryo is oriented with its anterior end at

the top of the screen. Dendra2red expressing hypoblast cells separate from the photoconverted region by 1 hr. These cells continue to migrate towards the anterior germinal crescent region until the time lapse is halted (4 hrs). The embryo moves out of focus during imaging, and thus imaging is halted and readjusted multiple times. The video shown here is a concatenated series of original videos acquired. Dorsal view. Scale:100 µm in all videos.

Imaging metadata:

Supplementary Movie 1

Microscope metadata: Images were captured using an Android Motorola E5 camera with a 0.7 MP camera. The phone was programmed to capture JPEG images at 5 min intervals, and the images were stored in the attached computer. These JPEGS were then processed into TIFF files or.avi files and annotated using NIH ImageJ 1.52t.

Supplementary Movie 2

Microscope metadata: Olympus MVX10 epifluorescence stereomicroscope (upright) equipped with a 3 filter turret with brightfield filter capable of holding DAPI (UV), CFP, GFP,YFP,Cy3 or Cy5 filter cubes. The 100W mercury apo lamp housing and transformer is used as the light source with a MVPLAPO 1x / 0.25NA objective and equipped with an Olympus XM10 camera controlled by the Olympus CellSens dimension software to acquire dynamic 2D images of the developing embryos.

Microscope metadata: Olympus MVX10 epifluorescence stereomicroscope with a MVPLAPO 1x / 0.25NA objective; filter ET - EGFP (FITC/Cy2); Ex ET470/40x; Em ET525/50m; Exposure time: 550 ms.

Dimensions: 8.9 mm X 6.7 mm. Resolutions: 0.15 pixels per µm. Voxel size: 6.45 x 6.45 μm^2. Bits per pixel: 16. Time frames: 60 Time intervals: 10 mins/frame acquired.

Supplementary Movie 3

Microscope metadata: Olympus MVX10 epifluorescence stereomicroscope (upright) equipped with a 3 filter turret with brightfield filter capable of holding DAPI (UV), CFP, GFP,YFP,Cy3 or Cy5 filter cubes. The 100W mercury apo lamp housing and transformer is used as the light source with a MVPLAPO 1x / 0.25NA objective and equipped with an Olympus XM10 camera controlled by the Olympus CellSens dimension software to acquire dynamic 2D images of the embryos.

Microscope metadata: Olympus MVX10 epifluorescence stereomicroscope with a MVPLAPO 1x / 0.25NA objective; Ex ET - Cy5; Ex: ET620/60x / Em: ET700/75m; Exposure time: 50 ms.

Dimensions: 8.9 mm X 6.7 mm. Resolutions: 0.15 pixels per µm. Voxel size: 6.45 x 6.45 μm^2. Bits per pixel: 16. Time frames:152 Time interval: 50ms/frame acquired.

Supplementary Movie 4

Microscope metadata: Zeiss LSM 780 (upright) equipped with 34 channel GaAsp Spectral Detectors,5 laser lines (405, 458, 488, 514, 561 and 633 nm), and run with ZEN 2011 system software was used to collect all the 3D images.

Photoconversion settings for Cardiac crest cell migration: 405nm laser: 10% scan speed: 6 (pixel dwell time: 6.30µsec) for 80 iterations. Photoconverted region: X Y: 126 µm X 530 µm.

Metadata for time lapse: Zeiss LSM 780 upright confocal microscope; W Plan-Apochromat 20x/1.0 DIC (UV) VIS-IR M27 75 mm objective; Zoom: 0.8x; Ex 458 (18%)/Em 446-543; Ex 561 (10.0%)/Em 446-543;Pixel dwell time, 1.58 µsec; mean of 4 frame scans. Dimensions: 1006.70 x 1009.81 x 195.00 μm^3 (39 z-sections) Resolution: 0.96 pixels per µm. Voxel size: 1.04 x 1.04 x 5 μm^3. Bits per pixel: 16

Supplementary Movie 5

Figure metadata: Zeiss LSM 780 (inverted) equipped with 34 channel GaAsp Spectral Detectors, 5 laser lines (405, 458, 488, 514, 561 and 633 nm), and run with ZEN 2011 system software was used to collect all the data. The microscope is equipped with an on-stage incubator which maintained the temperature at 37ºC during imaging and was used to acquire Movie 6.

Time-lapse: Dimensions: 5524.04 X 5507.43 X 1100.00 μm^3 (11 slices) Time points: 149, time interval: 10 mins. Resolution: 0.18 pixels per µm; Voxel size: 5.535 µm x 5.535 µm x 100 µm; Bits per pixel: 16. EC Plan-Neofluar 5x/0.16 M27. Zoom: 0.6x. Ex 488 (2%)/Em 491 - 588 (eGFP). Pixel dwell time:12.6 µsec; mean of 2 line scans.

Supplementary Movie 6

Figure metadata: Zeiss LSM 780 (inverted) equipped with 34 channel GaAsp Spectral Detectors, 5 laser lines (405, 458, 488, 514, 561 and 633 nm), and run with ZEN 2011 system software was used to collect all the data. The microscope is equipped with an on-stage incubator which maintained the temperature at 37ºC during imaging.

Time-lapse: Dimensions: 1346.14 X 1346.14 X 144.00 μm^3 (24 slices) Time points: 28, time interval: 10 mins. Resolution: 0.72 pixels per µm; Voxel size: 1.384 µm X 1.384 µm x 6.000 µm; Bits per pixel: 16. Plan-Apochromat 20x/0.8 M27. Zoom: 0.6x. Ex 458 (6%)/Em 446-544 (Cerulean). Ex 561 (2.6%-8%)/Em 562-695 (Kaede red). Pixel dwell time: 2.55 µsec; mean of 4 frame scans.
